# EspO1-2 Regulates EspM2-Mediated RhoA Activity to Stabilize Formation of Focal Adhesions in Enterohemorrhagic *Escherichia coli*-Infected Host Cells

**DOI:** 10.1371/journal.pone.0055960

**Published:** 2013-02-08

**Authors:** Tomoko Morita-Ishihara, Masashi Miura, Sunao Iyoda, Hidemasa Izumiya, Haruo Watanabe, Makoto Ohnishi, Jun Terajima

**Affiliations:** 1 Department of Bacteriology I, National Institute of Infectious Diseases, Shinjuku-ku, Tokyo, Japan; 2 Department of Eco-Epidemiology, Institute of Tropical Medicine Nagasaki University, Nagasaki-shi, Nagasaki, Japan; Friedrich-Alexander-University Erlangen-Nurenberg, Germany

## Abstract

Enterohemorrhagic *Escherichia coli* (EHEC) Sakai strain encodes two homologous type III effectors, EspO1-1 and EspO1-2. These EspO1s have amino acid sequence homology with *Shigella* OspE, which targets integrin-linked kinase to stabilize formation of focal adhesions (FAs). Like OspE, EspO1-1 was localized to FAs in EHEC-infected cells, but EspO1-2 was localized in the cytoplasm. An EHEC Δ*espO1-1*Δ*espO1-2* double mutant induced cell rounding and FA loss in most of infected cells, but neither the Δ*espO1-1* nor Δ*espO1-2* single mutant did. These results suggested that EspO1-2 functioned in the cytoplasm by a different mechanism from EspO1-1 and OspE. Since several type III effectors modulate Rho GTPase, which contributes to FA formation, we investigated whether EspO1-2 modulates the function of these type III effectors. We identified a direct interaction between EspO1-2 and EspM2, which acts as a RhoA guanine nucleotide exchange factor. Upon ectopic co-expression, EspO1-2 co-localized with EspM2 in the cytoplasm and suppressed EspM2-mediated stress fiber formation. Consistent with these findings, an Δ*espO1-1*Δ*espO1-2*Δ*espM2* triple mutant did not induce cell rounding in epithelial cells. These results indicated that EspO1-2 interacted with EspM2 to regulate EspM2-mediated RhoA activity and stabilize FA formation during EHEC infection.

## Introduction

Enterohemorrhagic *Escherichia coli* (EHEC) strains are important human pathogens, causing hemorrhagic colitis and hemolytic-uremic syndrome [1]–[3]. When EHEC colonizes the host intestine, it induces attaching and effacing (A/E) lesions. A/E lesions are characterized by loss of intestinal brush-border microvilli following intimate attachment of bacteria to intestinal epithelial cells. The characteristic actin condensation beneath the bacteria, resulting in formation of pedestal-like protrusions from the host cells, induces the intimate attachment [4]. The A/E lesions are dependent on delivery of bacterial virulence proteins, termed type III effectors, into host cells through a type III secretion system (T3SS). Type III effectors and the T3SS are highly conserved in many enteropathogenic bacteria. Some homologous type III effectors, found in EHEC, enteropathogenic *E. coli* (EPEC), *Citrobacter rodentium*, *Shigella* spp. and *Salmonella* spp., have been shown to have similar functions [5]–[7]. During infection, EHEC takes over various cell functions to facilitate bacterial colonization, multiplication and survival within the host by the use of type III effectors to reorganize the host cytoskeleton, modulate Rho GTPase signaling, inhibit apoptosis, and interfere with inflammatory signaling pathways and phagocytosis. Genes of T3SS and some type III effectors and their regulators in EHEC are encoded in a pathogenicity island termed the locus of enterocyte effacement (LEE) [1]–[3], [8], [9]. In addition, some type III effector genes are encoded at chromosomal loci outside the LEE and are termed non-LEE-encoded effectors (Nles) [8], [10]. The genetic structure and function of the LEE region are well-conserved in several intestinal pathogens that induce A/E lesions; i.e., EHEC, EPEC, *C. rodentium* [11].

The members of the Rho GTPase family act as key regulators to control the organization of the actin cytoskeleton in eukaryotic cells. Rho GTPases cycle between an active GTP- and inactive GDP-bound state. The Rho GTPase state is controlled by regulatory proteins, such as guanine nucleotide exchange factors (GEFs) that catalyze the exchange of GDP by GTP to activate Rho, guanine nucleotide dissociation inhibitors (GDIs) that inhibit GDP release to keep Rho inactive, and GTPase activating proteins (GAPs) that increase the rate at which Rho hydrolyzes GTP and consequently is inactivated. Activated Rho GTPase interacts with cellular effector proteins that induce reorganization of the actin cytoskeleton followed by changes in cell shape, cell polarity, cell movement, and cytokinesis [12], [13]. In addition, the Rho GTPase-effector protein interaction drives a large variety of biological responses, including transcriptional regulation, chemotaxis, axonal guidance, cell cycle progression, cell adhesion, oncogenic transformation, and epithelial wound repair [14]–[16] Several type III effectors of A/E pathogens can modulate Rho GTPases and their signaling pathways. A/E pathogen effectors Map, EspM and EspT modulate the signaling pathways of Cdc42, RhoA and Rac1/Cdc42 that trigger formation of filopodia, stress fibers and membrane ruffles/lamellipodia, respectively [17]–[19] In contrast to this direct modulation of Rho GTPase signaling pathways by Map, EspM and EspT, A/E pathogen effector EspG modulates Rho GTPase signaling pathways by indirectly modulating Rho GTPase signaling pathways. EspG homologs disrupt microtubule networks and induce release of a RhoA-specific guanine nucleotide exchange factor, GEF-H1, from the disrupted microtubules. The released GEF-H1 activates RhoA [20].

In this study, we investigated EspO1-1 and EspO1-2, which are OspE homologs in the EHEC Sakai strain. *Shigella* OspE targets integrin-linked kinase (ILK) at focal adhesions (FAs) to reinforce epithelial cell adherence to the extracellular matrix (ECM) [21]. Since EspO1-1 has limited amino acid similarity to EspO1-2, we investigated whether the EHEC OspE homologs might have different mechanisms for affecting host cell functions. Although EHEC EspO1-1 can localize at FAs in infected cells, EspO1-2 seems to be distributed in the cytoplasm. We investigated EspO1-2 localization, binding interactions and function in epithelial cells during infection with the EHEC Sakai strain.

## Results

### EspO1-1 and EspO1-2 Stabilize FAs and the Actin Cytoskeleton in EHEC-infected Cells

**Figure 1:**
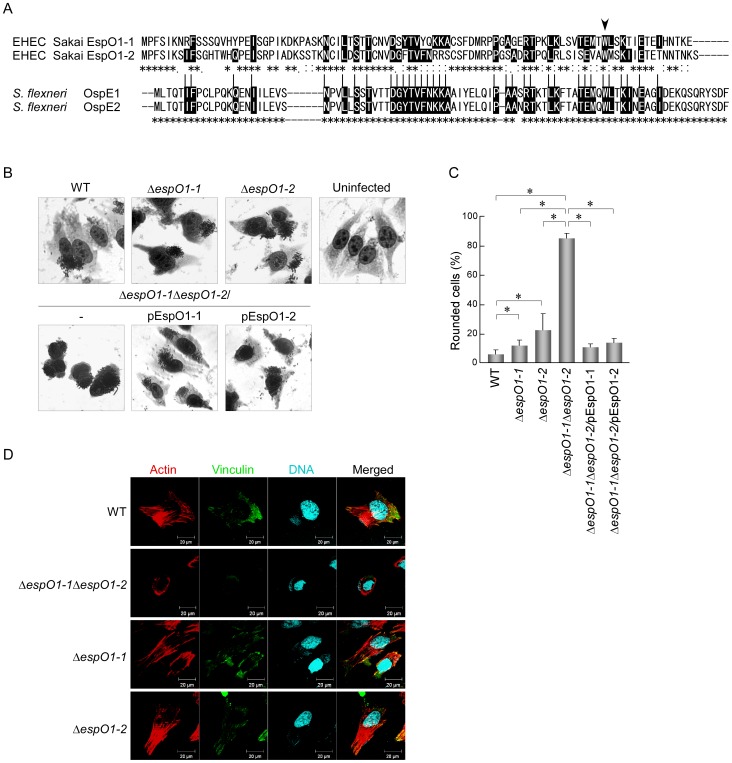
EspO1-1 and EspO1-2 stabilize FAs and the actin cytoskeleton in EHEC-infected cells. (A) Alignment of OspE2 homologs EspO1-1 and EspO1-2 in EHEC strain Sakai. EspO1-1 and EspO1-2 have 59% amino-acid sequence identity, and also have extensive homology with OspE2 (29% and 25% amino acid sequence identity to OspE2, respectively). Asterisks indicate amino acid residues that are conserved in either OspE1 and OspE2 or in EspO1-1 and EspO1-2. Black highlight indicates an amino-acid residue conserved in both OspE1 and OspE2 and either EspO1-1, EspO1-2, or both EspO1-1 and EspO1-2. Arrowhead indicates the tryptophan, which is involved in ILK binding, at amino acid residue 77 of EspO1s and residue 68 of OspEs. (B) Morphological change in HeLa cells uninfected or infected with EHEC. HeLa cells were infected with EHEC wild-type (WT), an Δ*espO1-1* mutant (Δ*espO1-1*), an Δ*espO1-2* mutant (Δ*espO1-2*), an Δ*espO1-1*Δ*espO1-2* double mutant (Δ*espO1-1*Δ*espO1-2*), an Δ*espO1-1*Δ*espO1-2*/pEspO1-1 or an Δ*espO1-1*Δ*espO1-2*/pEspO1-2. At 4 h post-infection, the cells were fixed and Giemsa-stained. (C) Percent cell rounding in cells infected by WT, Δ*espO1-1*Δ*espO1-2*, Δ*espO1-1* or Δ*espO1-2* shown in (B) and quantified (>50 cells, n≥3). Data are the mean and S.D. *P<0.001. The percent of cell rounding of uninfected cells was <3%. (D) Formation of actin filaments and focal adhesions (FAs) in HeLa cells infected with EHEC. HeLa cells were infected with WT, Δ*espO1-1*Δ*espO1-2*, Δ*espO1-1* or Δ*espO1-2*. At 4 h post-infection, the cells were fixed and processed for confocal laser scanning microscopy using rhodamine-phalloidin to visualize actin filaments (red), anti-vinculin antibody to visualize FAs (green), and DAPI to visualize DNA/chromosomes (blue).

A recent study showed that OspE, a *Shigella* type III effector, interacts with ILK to interfere with FA disassembly [21]. Several OspE homologs found in *Shigella*, *Salmonella* and EHEC strains were shown to have a similar function [21]. The EHEC Sakai strain secretes two OspE homologs, EspO1-1 and EspO1-2 (Fig. 1A). However, these two EspO1s might be functionally distinct from each other, and perhaps from the OspEs, because the amino acid sequence identity of the two EspO1s (59%) was much lower than that of the two *Shigella* OspEs (98%) (Fig. 1A). To investigate this idea, we first examined the effect of EspO1-1 and EspO1-2 on cell rounding of EHEC-infected cells, which involves FA disassembly and cell detachment from the culture-dish. Epithelial cells were infected with single and double deletion mutants of EHEC Sakai *espO1-1* and *espO1-2* for 4 h and then stained with Giemsa. Like the wild-type (WT) strain, the Δ*espO1-1* and Δ*espO1-2* single mutants and the Δ*espO1-1*Δ*espO1-2* double mutant adhered to epithelial cells and formed microcolonies (Fig. 1B). While WT-infected cells showed spread cell morphology like uninfected cells, cell rounding was induced in >80% of the Δ*espO1-1*Δ*espO1-2* double mutant-infected cells (Figs. 1B and C). In contrast, cell rounding of Δ*espO1-1* and Δ*espO1-2* single mutant-infected cells was induced in <30% of infected cells (Figs. 1B and C).

Based on these results, we examined the effect of EspO1-1 and EspO1-2 on FAs and the actin cytoskeleton in EHEC-infected cells. To visualize FAs and actin, infected cells were fluorescence-stained with anti-vinculin antibody and phalloidin, respectively. FAs were observed in cells infected with the WT strain, the Δ*espO1-1* mutant and the Δ*espO1-2* mutant, but were reduced in Δ*espO1-1*Δ*espO1-2* double mutant-infected cells (Fig. 1D and S1). In addition, although Δ*espO1-1* and Δ*espO1-2* single mutant-infected cells showed formation of actin filaments similar to WT-infected cells, Δ*espO1-1*Δ*espO1-2* double mutant-infected cells showed actin condensation and disruption of the actin cytoskeleton (Fig. 1D). These results indicated that both EspO1-1 and EspO1-2 additively stabilized FAs and the actin cytoskeleton to prevent detachment of EHEC-infected cells from the culture dish.

### EspO1-1 Localizes at FAs and EspO1-2 Localizes in the Cytoplasm of EHEC-infected Cells

**Figure 2:**
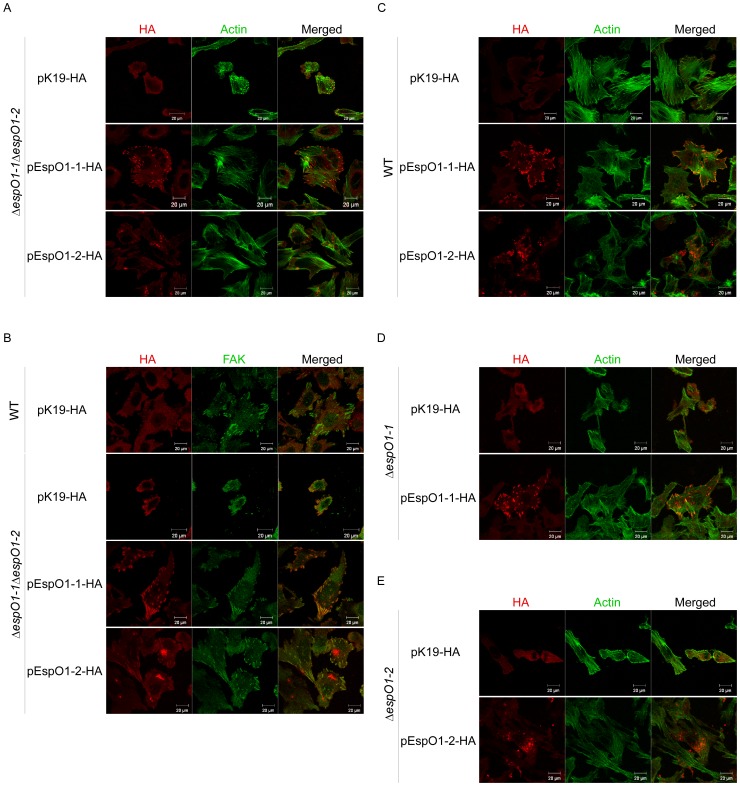
Localization of EspO1-1 and EspO1-2 in EHEC-infected cells. (A) HeLa cells were infected with Δ*espO1-1*Δ*espO1-2* carrying pK19-HA or Δ*espO1-1*Δ*espO1-2* carrying a plasmid with the gene for either HA-tagged EspO1-1 (pEspO1-1-HA) or EspO1-2 (pEspO1-2-HA) and, at 4 h post-infection, were analyzed by immunofluorescence staining. Cells infected with Δ*espO1-1*Δ*espO1-2* carrying pEspO1-1-HA or Δ*espO1-1*Δ*espO1-2* carrying pEspO1-2-HA were processed for confocal laser scanning microscopy using Alexa 488-conjugated phalloidin to visualize actin filaments (green), and anti-HA antibody to visualize HA-tagged proteins (red). (B) HeLa cells were infected with WT carrying pK19-HA, or Δ*espO1-1*Δ*espO1-2* carrying pK19-HA, pEspO1-1-HA or pEspO1-2-HA. Epithelial cells were infected with these strains for 4 h and immunofluorescence-stained with anti-FAK antibody to visualize focal adhesions (green) and anti-HA antibody (red). (C) HeLa cells were infected with WT carrying pK19-HA, pEspO1-1-HA or pEspO1-2-HA. (D) HeLa cells were infected with Δ*espO1-1* carrying pK19-HA or pEspO1-1-HA. (E) HeLa cells were infected with Δ*espO1-2* carrying pK19-HA or pEspO1-2-HA. Epithelial cells were infected with these strains for 4 h and immunofluorescence-stained with Alexa 488-conjugated phalloidin (green) and anti-HA antibody (red).

Although cell rounding of Δ*espO1-1* and Δ*espO1-2* single mutant-infected cells was induced in <30% of infected cells, the rate was greater than the rate of cell rounding of WT-infected cells (Fig. 1C). This result suggested that secretion of either EspO1-1 or EspO1-2 alone was not sufficient to attach infected cells to a culture dish. This suggested that EspO1-1 and EspO1-2 might target the same intracellular factor in EHEC-infected cells and, by interacting with the same intracellular factor, act cooperatively to stabilize FA formation and actin cytoskeleton structure. To examine this possibility, we tried to confirm that both EspO1 proteins were recruited to FA, the intracellular location where OspE acts in infected cells. An EHEC Δ*espO1-1*Δ*espO1-2* double mutant carrying plasmid pEspO1-1-HA or pEspO1-2-HA was used to determine the location of secreted HA-tagged EspO1-1 (EspO1-1-HA) or EspO1-2 (EspO1-2-HA), respectively, in infected cells (Figs. 2A and B). Immunoblot analyses with anti-HA antibody showed that these strains secreted equivalent amounts of EspO1-1-HA and EspO1-2-HA into the culture supernatant (data not shown). Epithelial cells were infected with these strains for 4 h and immunofluorescence-stained with anti-HA antibody (Figs. 2A and B). EspO1-1-HA accumulated strongly at the ends of actin filaments and at FA sites, as visualized by FAK staining, in the infected cells. However, EspO1-2-HA was scattered in the cytoplasm, not at the ends of actin filaments, and accumulated strongly at places other than the ends of actin filaments or FA sites in the infected cells. This indicated that EspO1-2 might function without FA localization in infected cells. This possibility was supported by the observation that cells infected with the Δ*espO1-1*Δ*espO1-2* double mutant carrying pEspO1-2-HA maintained a spread cell morphology without EspO1-1 (Figs. 2A and B). In addition, localization of both EspO1-1-HA and EspO1-2-HA was similar to that in cells infected with EHEC WT and the Δ*espO1-1* and Δ*espO1-2* single mutants carrying plasmid pEspO1-1-HA or pEspO1-2-HA (Figs. 2C, D and E). These results suggested that EspO1-1 could function at FAs in EHEC-infected cells as *Shigella* OspE does, and that EspO1-2 could function in the cytoplasm.

### EspO1-2 Binds Directly to the EspM2 Type III Effector

**Figure 3:**
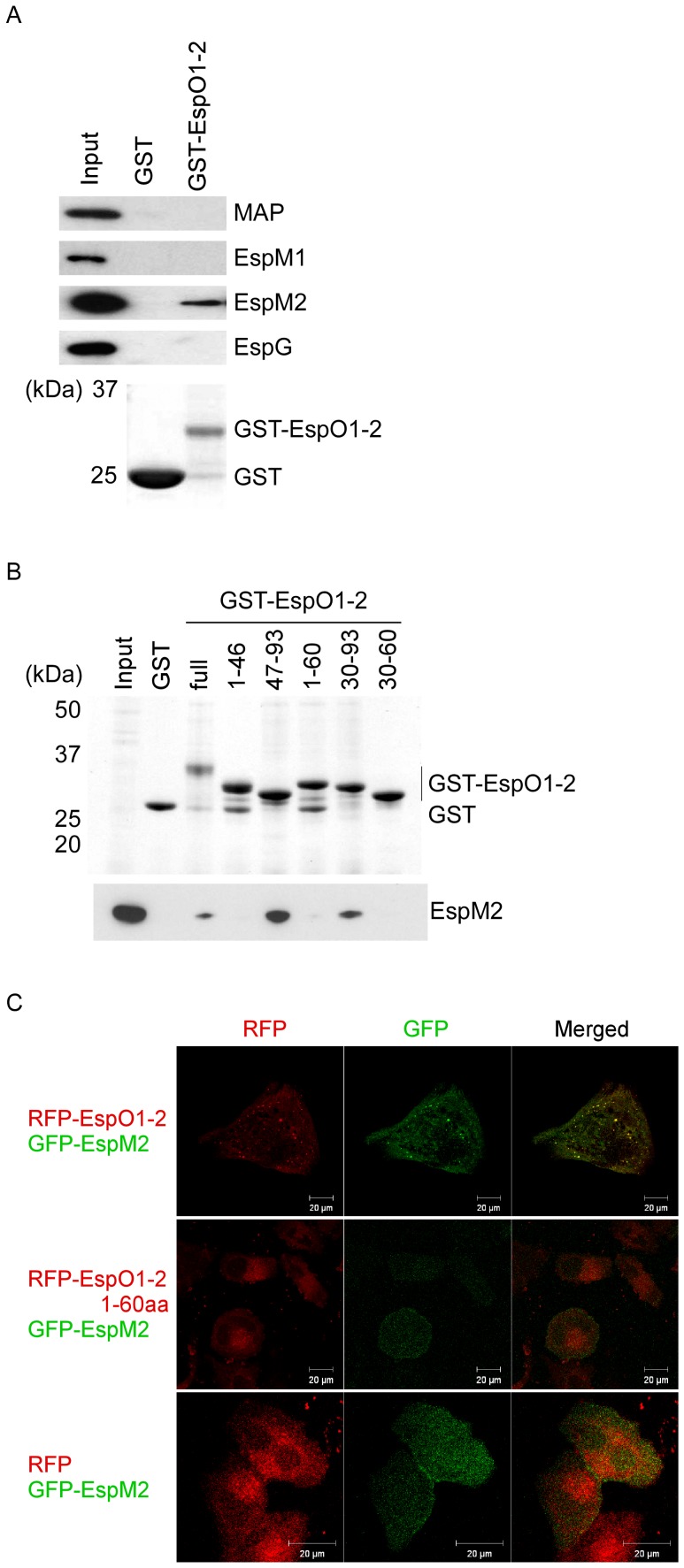
Interaction of EspO1-2 with EspM2. (A) GST-EspO1-2 binding assays were performed using cell lysates expressing Flag-tagged Map, EspM2, EspM2 or EspG. Proteins that bound to GST-EspO1-2 and GST-alone were analyzed by SDS-PAGE followed by immunoblotting with anti-Flag antibody. (B) To map the EspM2-binding site in EspO1-2, a series of truncated GST-EspO1-2 peptides was used in binding assays with cell lysate expressing Flag-tagged EspM2. (C) Co-localization of RFP-EspO1-2 and GFP-EspM2 in HeLa cells. HeLa cells were transfected with pRFP-EspO1-2, pRFP-EspO1-2 1–60aa or pRFP (red) and pEGFP-EspM2 (green).

OspE homologs in EHEC and *S. typhimurium* have been shown to interact with ILK *in vitro* and to localize at FAs after ectopic expression [21]. We confirmed that EspO1-1 and EspO1-2 bound to ILK *in vitro* (Fig. S2A), and that ectopically expressed EspO1-1 and EspO1-2 localized at FAs in uninfected cells (Fig. S2B). However, the increase in the number of FAs by EspO1-2 was less than that by EspO1-1 (Fig. S2C) and FA localization of EspO1-2 was small in the number compared with that of EspO1-1 (Fig. S2B). Since EspO1-2 secreted into EHEC-infected cells localized in the cytoplasm, EspO1-2 might act in the cytoplasm to stabilize FAs and actin filaments. This suggested that EspO1-2 might interact with a factor other than ILK. Since stabilization of FAs and actin filaments is regulated by a Rho GTPase signaling pathway, EspO1-2 may be involved in regulation of Rho GTPase signaling in EHEC-infected cells. EHEC delivers several type III effectors (e.g., MAP, EspM1, EspM2 and EspG) into host cells to modulate Rho GTPase [17], [18], [22], [23]. Therefore, we investigated whether EspO1-2 might modulate the function of these type III effectors to control Rho GTPase signaling during EHEC infection by testing whether EspO1-2 interacted directly with these type III effectors. Lysates of cells expressing Flag-tagged MAP, EspM1, EspM2 or EspG were used in binding assays with glutathione-S-transferase (GST)-tagged EspO1-2 (GST-EspO1-2). The proteins bound to GST-EspO1-2 were analyzed by immunoblotting with an anti-Flag antibody. The results showed that EspO1-2 bound to EspM2, but not to MAP, EspM1 or EspG (Fig. 3A). To further map the EspM2-binding site in EspO1-2, a series of truncated EspO1-2 peptides fused to GST was prepared and used in binding assays with cell lysate expressing Flag-tagged EspM2. The analysis showed that the EspO1-2 C-terminal region (residues 61–93) was required for binding to EspM2 (Fig. 3B).

To demonstrate the interaction between EspO1-2 and EspM2 in epithelial cells, we investigated the localization of ectopically expressed red fluorescent protein-tagged EspO1-2 (RFP-EspO1-2) and green fluorescent protein-tagged EspM2 (GFP-EspM2) in epithelial cells. When transfected into epithelial cells, RFP-EspO1-2 co-localized with GFP-EspM2 in the cytoplasm at 72 h post-transfection (Fig. 3C). Immunostaining with vinculin showed that RFP-EspO1-2 localized at FAs (Fig. S2B). In cells expressing RFP-EspO1-2 N-terminal region (1–60 aa) and GFP-EspM2, RFP-EspO1-2 1–60aa was not co-localized with GFP-EspM2 at 72 h post-transfection. GFP-EspM2 was distributed throughout the cytoplasm at 72 h post-transfection, as previously reported [23]. These results indicated that EspO1-2 interacted with EspM2 in the cytoplasm, rather than at FAs. We also did immunoprecipitation assays using cell lysate co-expressing GFP-EspM2 and RFP-EspO1-2, but were unable to confirm an interaction between GFP-EspM2 and RFP-EspO1-2 because no specific GFP-EspM2 or RFP-EspO1-2 band could be detected due to the presence of non-specific bands in the western blot assays.

### Interaction between EspO1-2 and EspM2 Inhibits Formation of Stress Fibers Induced by EspM2

**Figure 4:**
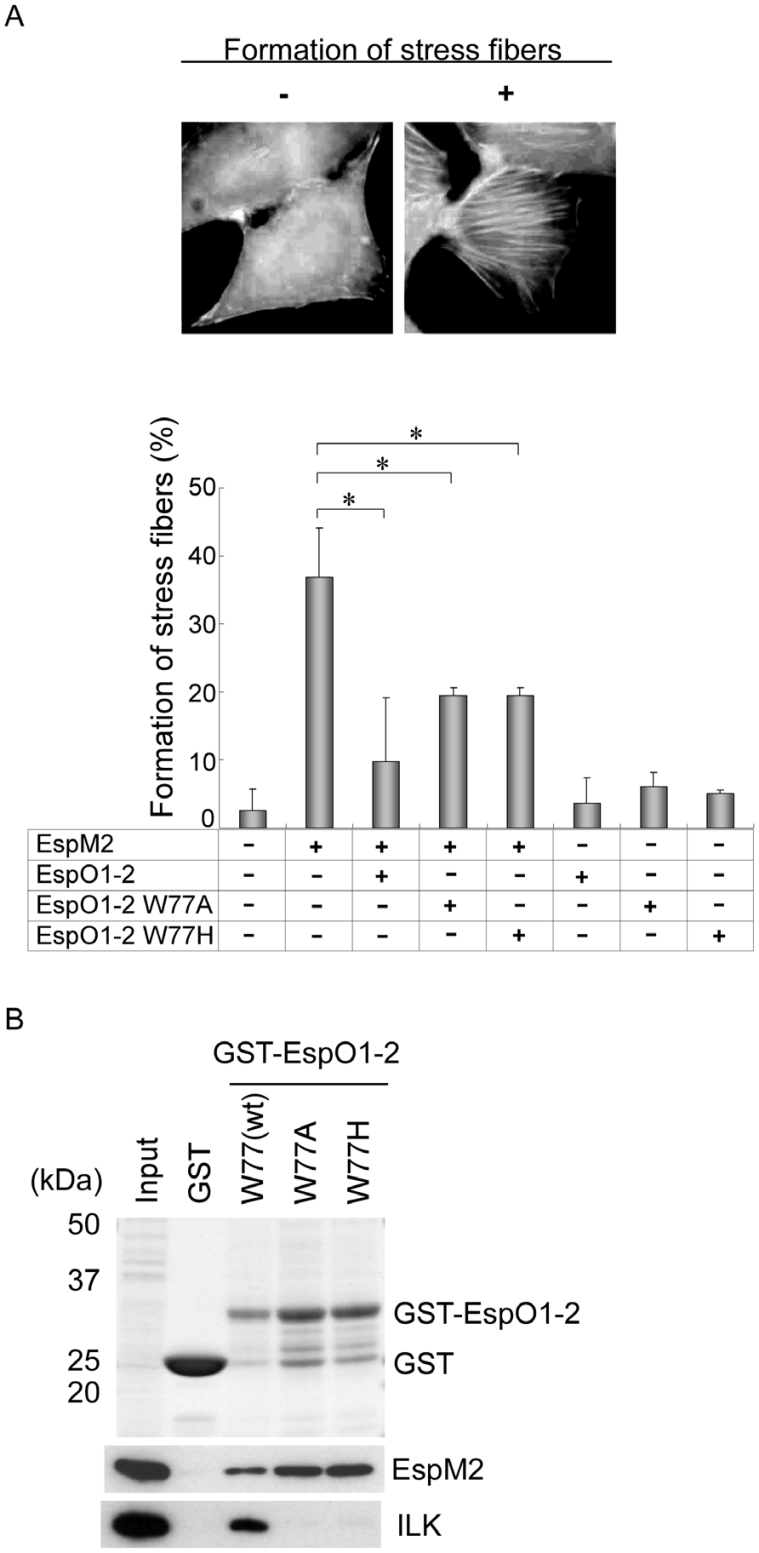
Interaction between EspO1-2 and EspM2 inhibits formation of stress fibers induced by EspM2. (A) HeLa cells transfected with pREP, pRFP-EspO1-2, pRFP-EspO1-2 W77A or pRFP-EspO1-2 W77H and pEGFP or pEGFP-EspM2 were immunostained with Alexa 350-conjugated phalloidin to visualize actin filaments and stress fibers (upper panels). Percent of transfected cells in which formation of dense stress fibers, as shown in the upper right panel, was induced (lower graph). (B) To investigate the effect of the EspO1-2 Trp77 residue on EspM2 binding, GST-EspO1-2 W77A and GST-EspO1-2 W77H, each with an amino acid substitution for EspO1-2 Trp77, were used in binding assays with cell lysate expressing Flag-tagged EspM2.

EspM2 has RhoA GEF activity and induces formation of stress fibers on both EHEC infection of host cells and ectopic expression of EspM2 [22]. To determine the functional significance of the interaction between EspO1-2 and EspM2 in epithelial cells, we investigated the effect of EspO1-2 on the formation of stress fibers induced by EspM2. Epithelial cells transfected with EspM2 and/or EspO1-2-expression vectors were fixed at 72 h post-transfection and stained with rhodamine phalloidin to visualize actin (Fig. 4A, upper panels) and the formation of stress fibers was determined as previously reported [22]. Stress fiber formation was induced in 38% of cells transfected with only the EspM2-expression vector (Fig. 4A, lower graph). However, formation of stress fibers decreased to approximately 10% of cells transfected with both EspM2 and EspO1-2 expression vectors. The formation of stress fibers in cells transfected with only the EspO1-2-expression vector was almost the same as in mock-transfected cells. These results indicated that EspO1-2 suppressed the formation of stress fibers induced by EspM2 in epithelial cells.

In addition, we tried to eliminate the effect of interaction between EspO1-2 and host ILK on formation of stress fibers. An OspE mutant with an amino-acid substitution for tryptophan (Trp) at residue 68 is unable to interact with ILK and localize to FAs [21]. Based on the sequence alignment, the Trp amino acid is conserved in the C-terminal region of EspO1-2 at residue 77 (Trp77) (Fig. 1A, arrowhead). Therefore, to first examine the effect of EspO1-2 Trp77 on interaction with EspM2, interaction assays were done using GST-EspO1-2 with an alanine (W77A) or histidine (W77H) substitution at EspO1-2 Trp77 (Fig. 4B). The assays made no distinction between direct and indirect interactions. EspO1-2 W77A and W77H did not interact with ILK, in agreement with a previous report [21]. However, the mutants did interact with EspM2 (Fig. 4B). This suggests that EspM2 and ILK may interact with a different region of EspO1-2. Next, we examined the effect of EspO1-2 W77A and W77H on formation of stress fibers using epithelial cells transfected with EspM2 and/or EspO1-2 W77A or W77H-expression vectors. The formation of stress fibers in cells transfected with EspM2 and either EspO1-2 W77A or W77H-expression vectors decreased to about 20% of the transfected cells (Fig. 4A, lower graph). Taken together, these results suggested that EspO1-2 interacted with EspM2 and inhibited the GEF activity of EspM2 that activated the RhoA signaling pathway.

### Enhancement of EspM2-mediated RhoA Signaling Activity Induces Cell Rounding

**Figure 5:**
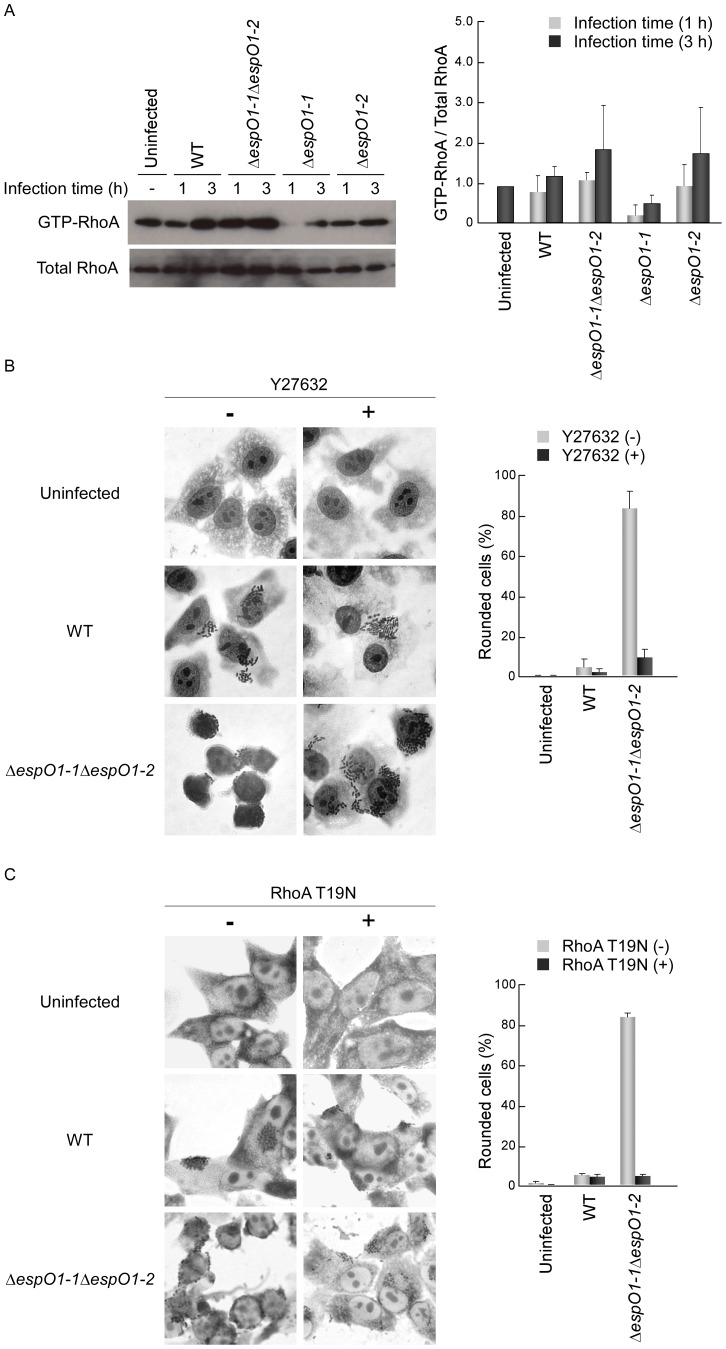
Enhancement of RhoA signaling activity induces cell rounding. (A) Involvement of EspO1-1 and EspO1-2 in the RhoA-ROCK signaling pathway in EHEC-infected cells. HeLa cells were infected with the EHEC WT, an Δ*espO1-1*Δ*espO1-2* double mutant, an Δ*espO1-1* mutant or an Δ*espO1-2* mutant. At 1 and 3 h post-infection, a sample of cells was lysed and GTP-RhoA, the active form of RhoA, was co-precipitated with the GST-Rho binding domain (RBD) of rhotekin. The amount of RhoA bound to RBD (GTP-RhoA) and total RhoA in each cell lysate was analyzed by immunoblotting with anti-RhoA antibodies. The intensity of bands was quantified using the Image J program, and the results are expressed as fold increases in the ratio for GTP-RhoA/Total RhoA in uninfected cells (n = 3). (B) Effect of the ROCK inhibitor Y27632 on the morphological change of EHEC-infected cells. Untreated HeLa cells and HeLa cells treated with Y27632 for 1 h were infected with EHEC WT or the Δ*espO1-1*Δ*espO1-2* double mutant. At 3 h post-infection, the cells were fixed and stained with Giemsa. The percent of cell rounding in cells infected by WT or Δ*espO1-1*Δ*espO1-2* was quantified (>50 cells, n≥3). (C) Effect by expression of dominant-negative RhoA (T19N) in transfected cells on the morphological change of EHEC-infected cells. HeLa cells transfected with GFP or GFP-tagged RhoA T19N were infected with EHEC WT or the Δ*espO1-1*Δ*espO1-2* double mutant at 48 h post-transfection. At 3 h post-infection, the cells were fixed and stained with Giemsa. The percent of cell rounding in cells infected by WT or Δ*espO1-1*Δ*espO1-2* was quantified (>50 cells, n≥3).

Epithelial cells infected by EHEC with an *espO1-2* deletion were expected to show enhancement of RhoA signaling activity due to the sustained RhoA GEF activity of EspM2. Therefore, we examined the level of RhoA activity in cells infected with the Δ*espO1-1*Δ*espO1-2* double mutant and the Δ*espO1-1* and Δ*espO1-2* single mutants. Epithelial cells were infected with the EHEC WT, Δ*espO1-1*Δ*espO1-2* double mutant or Δ*espO1-1* and Δ*espO1-2* single mutants, and at 1 and 3 h post-infection an active form of RhoA (GTP-RhoA) in the lysates of infected cell samples was precipitated with the Rho binding domain (RBD) of rhotekin, which only binds GTP-RhoA. The amount of GTP-RhoA and total RhoA were determined by immunoblotting with anti-RhoA antibody. The amount of GTP-RhoA in WT-infected cells was comparable to that of uninfected cells at 1 h post-infection and increased at 3 h post-infection (Fig. 5A): the 3 h result was in agreement with that previously reported [20]. The amount of GTP-RhoA in the Δ*espO1-1*Δ*espO1-2* double mutant-infected cells at 1 h post-infection was comparable to that in WT- and uninfected cells and was greater than that in WT-infected cells at 3 h post-infection (Fig. 5A). Therefore, the increase in RhoA signaling activity occurred significantly after the 1 h of EHEC infection in Δ*espO1-1*Δ*espO1-2* double mutant-infected cells rather than in WT-infected cells. The amount of GTP-RhoA in the Δ*espO1-1* single mutant-infected cells at 1 and 3 h post-infection was lower than that in WT- and uninfected cells at 1 and 3 h post-infection. The amount of GTP-RhoA in the Δ*espO1-2* single mutant-infected cells at 1 h post-infection was greater than that in the Δ*espO1-1* single mutant-infected cells at 1 and 3 h post-infection and comparable to that in WT- and Δ*espO1-1*Δ*espO1-2* double mutant-infected cells at 1 h post-infection. The amount of GTP-RhoA in the Δ*espO1-2* single mutant-infected cells at 3 h post-infection was comparable to that in Δ*espO1-1*Δ*espO1-2* double mutant-infected cells at 3 h post-infection. These results indicate that the amount of the GTP-RhoA in the Δ*espO1-1*Δ*espO1-2* double mutant-infected cells at 1 and 3 h post-infection might be influenced by the absence of EspO1-2 rather than EspO1-1.

Since cell rounding was induced in Δ*espO1-1*Δ*espO1-2* double mutant-infected cells (Fig. 1B), we examined whether the increase in RhoA signaling activity in double mutant-infected cells played a role in cell rounding. Therefore, we treated epithelial cells with Y27632, a specific inhibitor of ROCK (a downstream effector of RhoA), to block RhoA-ROCK signaling activity in infected cells. Epithelial cells were treated with Y27632 for 1 h, infected with the Δ*espO1-1*Δ*espO1-2* double mutant, and at 4 h post-infection were fixed and Giemsa-stained. Y27632 treatment had no affect on bacterial attachment to epithelial cells or on the formation of bacterial microcolonies (Fig. 5B). However, cell rounding was not induced in Δ*espO1-1*Δ*espO1-2* double mutant-infected cells treated with Y27632 (Fig. 5B). This result indicated that Y27632 blocked the RhoA signaling that was activated in Δ*espO1-1*Δ*espO1-2* double mutant-infected cells. In order to examine whether RhoA is involved in cell rounding, we used epithelial cells transfected with dominant-negative RhoA (T19N). The cells at 48 h post-transfection were infected with the Δ*espO1-1*Δ*espO1-2* double mutant for 4 h. Expression of RhoA T19N was confirmed by increased GFP-tagged RhoA T19N using fluorescent confocal microscopy (data not shown). Cell rounding was not induced in Δ*espO1-1*Δ*espO1-2* double mutant-infected cells expressing RhoA T19N (Fig. 5C). Therefore, these results showed that RhoA signaling activity contributed to cell rounding.

### EspO1-2 Regulates the EspM2-mediated RhoA Signaling Pathway during EHEC Infection

**Figure 6:**
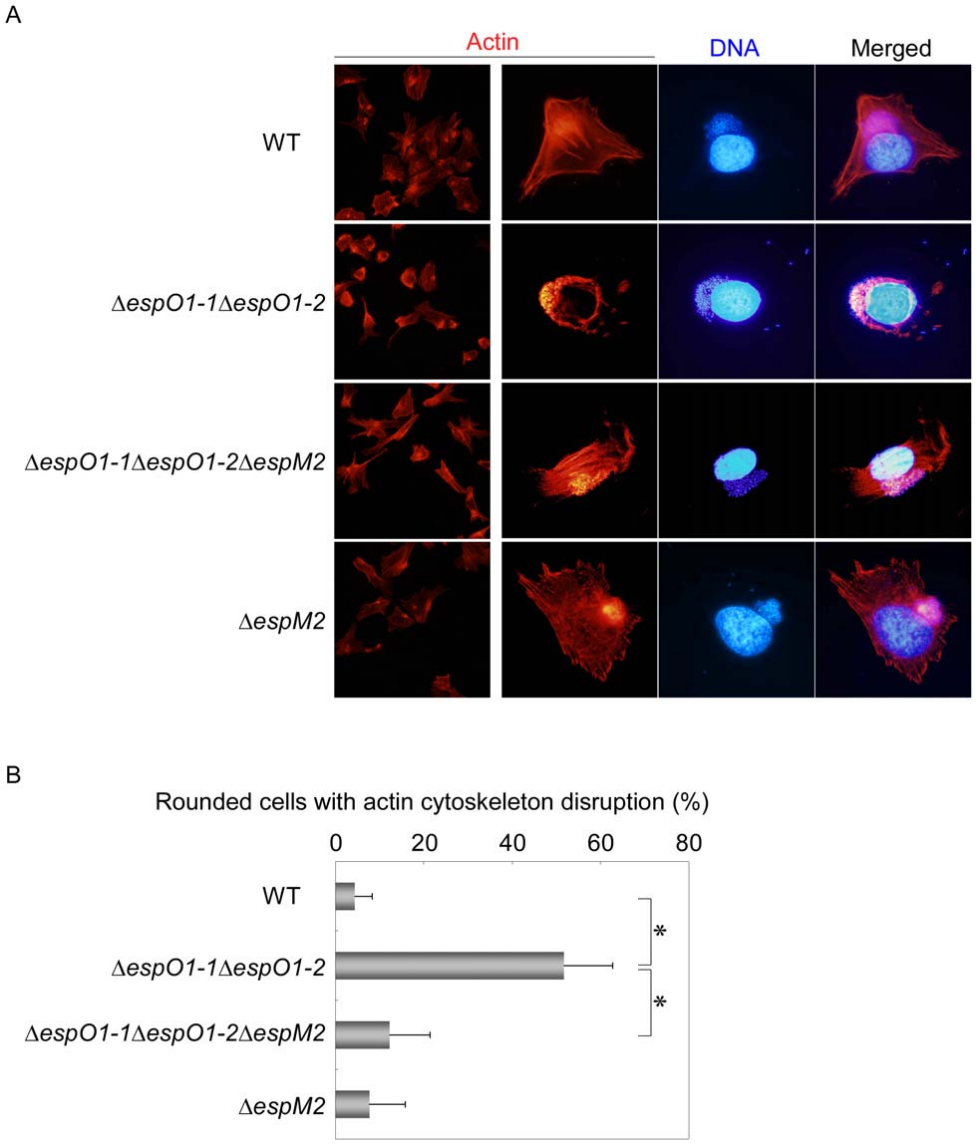
Contribution of EspM2 to cell rounding and actin cytoskeleton structure disruption in the infected cells. (A) HeLa cells were infected with an Δ*espO1-1*Δ*espO1-2*Δ*espM2* triple mutant and, at 4 h post-infection, stained with rhodamine-phalloidin to visualize actin filaments. (B) The percent of cells shown in (A) in which cell rounding was induced and the actin cytoskeleton was disrupted was quantified (>50 cells, n≥3). Data are the mean and S.D. *P<0.001.

**Figure 7:**
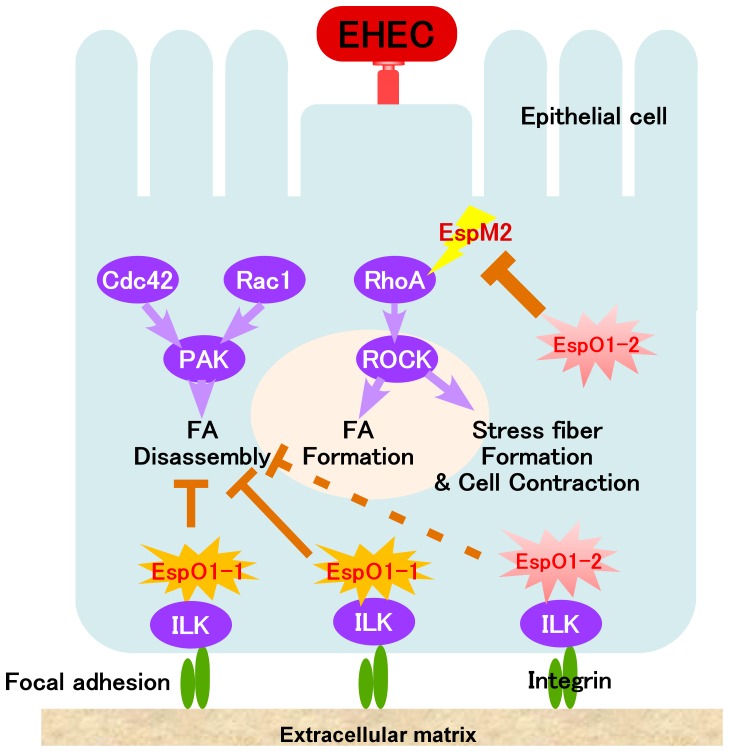
A proposed model for the role of EspO1-2 in an EHEC-infected cell. EspO1-2 interacts with EspM2 in EHEC-infected cells to suppress EspM2-enhanced host RhoA activity.

Since EspM2 activates the RhoA signaling pathway in EHEC-infected cells [22], EspM2 might act as a trigger for cell rounding in Δ*espO1-1*Δ*espO1-2* double mutant-infected cells. To examine the contribution of EspM2 to cell rounding, epithelial cells were infected with an Δ*espM2* single mutant or the Δ*espM2*Δ*espO1-1*Δ*espO1-2* triple mutant and cell morphology was examined at 4 h post-infection. Both mutants adhered to epithelial cells and formed dense microcolonies on the cell surface, similar to the EHEC WT and Δ*espO1-1*Δ*espO1-2* double mutant strains (Fig. 6A). Since cells that were rounded by infection with the Δ*espO1-1*Δ*espO1-2* double mutant showed disruption of filamentous actin and of the actin cytoskeleton structure (Fig. 1D), the Δ*espM2* and Δ*espM2*Δ*espO1-1*Δ*espO1-2* triple mutant-infected cells were stained with rhodamine phalloidin to visualize actin. Cell rounding was induced in 52% of Δ*espO1-1*Δ*espO1-2* double mutant-infected cells and the actin cytoskeleton structure was disrupted (Figs. 6A and B). However, only 12% of Δ*espM2*Δ*espO1-1*Δ*espO1-2* triple mutant-infected cells were rounded. This indicated that the triple mutant infection inhibited cell rounding and actin cytoskeleton structure disruption (Figs. 6A and B). Cell rounding and actin cytoskeleton structure disruption were not induced in Δ*espM2* mutant-infected cells (Fig. 6A). These results together suggested that EspO1-2 interacted with EspM2 to control EspM2-mediated RhoA signaling activity, thereby maintaining cell morphology and preventing cell detachment from the extracellular matrix by stabilizing FAs and the actin cytoskeleton during EHEC infection (Fig. 7).

## Discussion

In this study, we demonstrated a novel function for EHEC Sakai strain EspO1-2, a homolog of *Shigella* OspE. We also showed that EspO1-1 had a similar function to OspE. This was consistent with a recent finding that OspE homologs in EHEC, EPEC, *C. rodentium*, *Shigella* spp. and *Salmonella* spp. interact with ILK and interfere with FA disassembly [21]. However, EspO1-2 was found to interact with EspM2 to modulate host RhoA activity in EHEC-infected cells. EspM2 has Rho GEF activity and is involved in RhoA activation, pedestal formation and tight junction localization in EHEC-infected cells [22]. Our results showed that EspO1-2 inhibited EspM2-mediated activation of host RhoA signaling. In host epithelial cells, activated RhoA promotes disruption of cell polarity and the epithelial barrier followed by epithelial cell detachment from the intestine [24]. Although the biological significance of EspM2 function in EHEC-infected cells remains unknown, activation of RhoA by EspM2 in EHEC-infected epithelial cells could result in serious disruption of host intestinal epithelia and development of pathogenic sequelae. This suggestion was supported by a report that a murine infection model for A/E pathogens, a highly susceptible CH3/HeJ mouse strain, showed that a *C. rodentium espM2* mutant was shed at significantly lower levels and caused less mortality than wild-type *C. rodentium*, indicating that EspM2 contributes to *C. rodentium* virulence [25]. In host defense against bacterial infection, disruption of host intestinal epithelia induced by bacterial infection promotes elimination of bacteria and prevents infection from spreading in the host. Our results suggested that secreted EspO1-2 interacted with EspM2 in infected cells to minimize disruption of host cell functions induced by infection, while EHEC created a host cell environment suitable for colonization. Furthermore, our results indicated that a type III effector might interact with another type III effector to modulate the effector’s function in infected cells, although a large majority of experimentally analyzed type III effectors target host proteins to modulate their functions in host cells.

A number of bacterial effectors or toxins capable of modulating the host Rho pathway have been reported. A recent study showed that a type III effector targets host Rho GEF to modulate the Rho GTPase signaling pathway (Dong et al., 2010). EspH binds directly to the Dbl homology-pleckstrin homology (DH-PH) domain in several Rho GEFs. The DH-PH domain catalyses Rho GTPase nucleotide change. EspH bound to the DH-PH domain prevents GEF binding to Rho GTPase, thereby inhibiting nucleotide exchange-mediated Rho GTPase activation [26]. EspM2 does not have sequence homology to a DH-PH domain. There have been no reports of EspH bound to EspM2 so far. In general, a Rho GEF-Rho GTPase complex is dissociated by GTP or GDP. However, such exogenous nucleotides do not dissociate the EspM2-RhoA complex [17], [18], [22], [23]. These results indicated that EspM2 bound to RhoA by a unique mechanism and that the function of the EspM2-RhoA complex was not blocked by GTP-GDP exchange. Therefore, the interaction between EspO1-2 and EspM2 appeared to play an important role in a bacterial novel mechanism for controlling the RhoA activity.

We showed that cell rounding of Δ*espO1-1*Δ*espO1-2* double mutant-infected cells was induced by EspM2-mediated RhoA activity. Disruption of the actin cytoskeleton and loss of FAs were observed in many rounded infected cells. Stress fibers and FAs are known to be functionally interactive structures [27]–[31]. FAs initiate the elongation of stress fibers [28]. The formation of stress fibers connected to FAs organizes contractile actomyosin bundles and actin assembly in response to RhoA [32], [33]. The tension generated by stress fibers enhances the growth of mechanosensitive FAs. Interestingly, high RhoA activity and increased actomyosin contractility have been reported to be an important ‘trigger’ for promoting FA disassembly at the cell trailing edge [34]. EspM2-mediated Rho activity can enhance the formation of stress fibers. Hence, FA growth should increase in rounded infected cells, but cell rounding actually induced loss of FAs in this study. This result indicated that EspM2-mediated RhoA activity may have resulted in enhanced growth of FAs, increased contractility, and rapid FA turnover with FA disassembly. Therefore, EspM2 acted as one of the triggers for cell rounding in Δ*espO1-1*Δ*espO1-2* double mutant-infected cells. During EspM2-dependent FA disassembly, the absence of EspO1-1 should also promote FA disassembly because EspO1-1 can interfere with FA disassembly by targeting ILK. This hypothesis is supported by the finding that cell rounding decreased in Δ*espO1-2* mutant-infected cells compared to Δ*espO1-1*Δ*espO1-2* double mutant-infected cells. We also confirmed that the partial morphological change in Δ*espO1-2* mutant-infected cells looked like cell contraction induced by activation of the RhoA-ROCK signaling pathway. The morphological change might be caused by contractile actomyosin bundles induced by EspM2-mediated RhoA activity. In addition, ectopically-expressed EspM2 also appeared to have induced cell contraction in most epithelial cells. Δ*espO1-1* mutant-infected cells also showed a similar morphological change. Although the detailed mechanism for the morphological change is unclear, it could be due to FA disassembly induced by the absence of EspO1-1 [21]. Since EspG, a type III effector, can activate host RhoA by microtubule disruption followed by release of GEF-H1, actomyosin contractility also might increase in Δ*espO1-1* mutant-infected cells in an EspM2-independent manner.

We also found that EspO1-2 has the potential to interact with ILK to interfere with FA disassembly. In an experiment using transfection of epithelial cells with EspO1-2, ectopically-expressed EspO1-2 targeted FA and increased the number of FAs (Figs. S2B and C). However, the increase in the number of FAs by EspO1-2 was less than that by EspO1-1 (Fig. S2C), although EspO1-1 and EspO1-2 did not have a significant difference in affinity for ILK (Fig. S2A). Since localization of EspO1-2 at FAs was observed in cells with ectopically expressed EspO1-2 but not in infected cells (Figs. 2 and S2B), EspO1-2 may have the means of using its effect on other yet unidentified EHEC effectors or host proteins to interact with EspM2 rather than with ILK during EHEC infection. Moreover, we considered the possibility that the interaction between EspO1-2 and EspM2 causes less efficient localization of EspO1-2 at FAs, and investigated localization of EspO1-2 in EHEC-infected cells in the absence or presence of EspM2. In the Δ*espM2*Δ*espO1-1*Δ*espO1-2* triple mutant-infected cells efficient localization of EspO1-2 at FAs slightly-increased (Fig. S3A), but there were no significant differences in FA localization of EspO1-2 in the Δ*espM2*Δ*espO1-1*Δ*espO1-2* triple mutant and the Δ*espO1-1*Δ*espO1-2* double mutant-infected cells (Fig. S3B). Additionally, we confirmed that EspO1-1 is also able to interact with EspM2 because of having similar amino acid sequences to EspO1-2 (Fig. S2D). However, compared to EspO1-2, EspO1-1 showed a decline in the interaction ability between its C-terminal region and EspM2 (Fig. S2D). Thus, these two EspO1s may have different mechanisms for affecting host cell functions in spite of having the potential to interact with both EspM2 and ILK.

OspE homologs are widely conserved in enteric bacterial pathogens; e.g., EPEC Esp1, *C. rodentium* EspO1, *Shigella* spp. OspE1 and OspE2, and *Salmonella* spp. Osp1 [21]. Two OspEs in *S. flexneri* are functionally-indistinguishable and have 98% amino acid identity and 100% similarity (Fig. 1A) [21]. However, in this study, two OspE homologs in EHEC Sakai were found to have different functions. Based on the complete genome sequences in EHEC O157 Sakai, O26 11368 and O111 11128 strains [35], the amino acid sequences of the C-terminal region of the OspE homologs in these strains, which corresponds to an EspM2 binding region, were aligned. Each strain has two OspE homologs with limited amino acid identity (Fig. S4). The amino acid sequence analysis classified the OspE homologs into two groups, one similar to EspO1-1 and the other similar to EspO1-2 (Fig. S4). Therefore, EHEC may utilize two types of EspOs during infection to modulate the host cytoskeleton and to create micro-environments suitable for colonization.

## Materials and Methods

### Bacterial Strains, Plasmids, Cell Culture, and Media

The bacterial strains and plasmids used in this study are listed in Table 1. SKI-5142 is a *lac*-negative derivative of the EHEC O157:H7 Sakai strain [36] and was used as the wild-type strain in this study. The following deletion mutants were constructed from strain SKI-5142 using a one-step inactivation method [37]: SKI-5400S and SKI-5403S were Δ*espO1-1* and Δ*espO1-2* single deletion mutants, respectively; SKI-5401S was an Δ*espO1-1*Δ*espO1-2* double deletion mutant; SKI-5404 was an Δ*espM2* single deletion mutant; and SKI-5402 was an Δ*espO1-1*Δ*espO1-2*Δ*espM2* triple deletion mutant. PCR primer set ECs1567-P1/ECs1567-P2 (Table 2) was used to construct strain SKI-5400S and to insert Δ*espO1-1* to construct an Δ*espO1-1* single deletion mutant strain. PCR primer set ECs1821-P1/ECs1821-P2 (Table 2) was used to construct SKI-5403S, SKI-5401S and SKI-5402S and to insert Δ*espO1-2*, Δ*espO1-1/*Δ*espO1-2* or Δ*espO1-1*/Δ*espO1-2*/Δ*espM2* to construct deletion mutant strains. PCR primer set Ecs3485-P5/Ecs3485P6 (Table 2) was used to construct SKI-5404 and SKI-5402 and to insert Δ*espM2* or Δ*espO1-1*/Δ*espO1-2*/Δ*espM2* to construct deletion mutant strains. The EHEC strains were grown in Dulbecco's Modified Eagle Medium (DMEM) containing 0.5% glycerol or on LB plates. The antibiotic concentrations were as follows: chloramphenicol (Cm), 25 µg/ml; ampicillin (Ap), 100 µg/ml; kanamycin (Km), 50 µg/ml.

**Table 1:** Bacterial strains and plasmids used in this study.

	Genotype or relevant phenotype	Source or reference
SKI-5142	EHEC O157:H7 Sakai Δ(*lacZYAI*)	[39]
SKI-5400S	SKI-5142 Δ(*espO1-1*)	This study
SKI-5403S	SKI-5142 Δ(*espO1-2*)	This study
SKI-5401S	SKI-5400S Δ(*espO1-2*)	This study
SKI-5404	SKI-5401S Δ*(espM2*)::*kan*	This study
SKI-5402	SKI-5142 Δ(*espM2*)::*kan*	This study
pTH18cr	pSC101-*ori*, cloning vector; Cm^r^	[36]
pEspO1-1	pTH18cr derivative carrying the *espO1-1* gene with the *lac* promoter	This study
pEspO1-2	pTH18cr derivative carrying the *espO1-2* gene with the *lac* promoter	This study
pIRES-hrGFP-2a	cloning vector with a hemagglutinin (HA) tag gene; Ap^r^	Stratagene
pIRES-hrGFP-2a-*espO1-1*	pIRES-hrGFP-2a derivative carrying the *espO1-1* gene	This study
pIRES-hrGFP-2a-*espO1-2*	pIRES-hrGFP-2a derivative carrying the *espO1-2* gene	This study
pK19	pMB1-*ori*, cloning vector; Km^r^	[40]
pK19HA	pK19 derivative carrying the HA gene with the *lac* promoter	This study
pEspO1-1-HA	pK19 derivative carrying the *espO1-1*-HA gene with the *lac* promoter	This study
pEspO1-2-HA	pK19 derivative carrying the *espO1-2*-HA gene with the *lac* promoter	This study
pDsRed-monomer C1	Mammalian expression vector with a red fluorescence protein (RFP) gene; Km^r^, Neo^r^	Clontech
pREP-EspO1-1	pDsRed-monomerC1 derivative carrying the *espO1-1* gene	This study
pREP-EspO1-2	pDsRed-monomerC1 derivative carrying the *espO1-2* gene	This study
pEGFP-C1	Mammalian expression vector with a green fluorescent protein (EGFP) gene; Km^r^, Neo^r^	Clontech
pEGFP-EspM2	pEGFP-C1 derivative carrying the *espM2* gene	This study
pGEX-5X-1	Expression vector of Glutathione S-transferase (GST)-fused proteins with the tac promoter; Apr	GE Healthcare
pGST-EspO1-1	pGEX-5X-1 derivative carrying the *espO1-1* gene	This study
pGST-EspO1-2	pGEX-5X-1 derivative carrying the *espO1-2* gene	This study
pFLAG-MAC	Expression vector of FLAG-tagged proteins with the tac promoter; Apr	Sigma
pFLAG-EspM1	pFLAG-MAC derivative carrying the *espM1* gene	This study
pFLAG-EspM2	pFLAG-MAC derivative carrying the *espM2* gene	This study
pFLAG-MAP	pFLAG-MAC derivative carrying the *map* gene	This study
pFLAG-EspG	pFLAG-MAC derivative carrying the *espG* gene	This study

**Table 2:** Primer sequences used in this study.

Primer name	Primer sequence	Source or reference
ECs1821-P2	GAAGTTATTCATGGCTAGTGTACCAACTCTTTTCTGGGCTAATAGCATATGAATATCCTCCTTAGT	This study
ECs1821-P1	ACATTAAATTGCACTATAAGAGAAAGACATAATGTGAGGATAAAAGTGTAGGCTGGAGCTGCTTC	This study
ECs1567-P2	GGTGTTTCGGGTCTGTGGCTTTTTGTGTTCTCTGGTAAGTGGTTGCATATGAATATCCTCCTTAGT	This study
ECs1567-P1	ACGGAATAGCATAAAAACACTTTTCATGGAGCAAAGGAGAAAACAGTGTAGGCTGGAGCTGCTTC	This study
ECs3485-P5	ACTGGTCCGAAGCCGATGATGCTGAAGCAGTCCTCTCCTCTAGCCCTTACGCCCCGCCCTGCCAC	This study
ECs3485-P6	CAAGTATGCAATATTGTAAACACTTTTTTATAAAAAGGAATTATACTACCTGTGACGGAAGATCA	This study
IRES-XbaI-ds-1	ATTCATCTGCAGCCGCTCTAGCCCGGGCGGATCCG	This study
IRES-PstI-us-1	ATTCATTCTAGACCTATGCAGTCGTCGAGGAATTGC	This study
ECs1567-EcoRI-1-us	ATTCATGAATTCATATAAATATTCTTAATGATACCAAC	This study
ECs1567-SalI-2-ds	ATTCATGTCGACCACCTCCTGTGGAGGTGGTTGCTTGC	This study
ECs1821-EcoRI-1-us	ATTCATGAATTCTTTTTAGATTCATAGTTTAATGTTTC	This study
ECs1821-SalI-2-ds	ATTCATGTCGACGTTGTTAGGAAGTTATTCATGGCTAG	This study
IRES-XbaI-ds-1	ATTCATCTGCAGCCGCTCTAGCCCGGGCGGATCCG	This study
IRES-PstI-us-1	ATTCATTCTAGACCTATGCAGTCGTCGAGGAATTGC	This study
IRES-XbaI-ds-1	ATTCATCTGCAGCCGCTCTAGCCCGGGCGGATCCG	This study
IRES-PstI-us-1	ATTCATTCTAGACCTATGCAGTCGTCGAGGAATTGC	This study
ECs1567-EcoRI-2us	ATTCATGAATTCAAAACACTTTTCATGGAGCAAAGGAG	This study
ECs1567-SalI-1'-ds	ATTCATGTCGACCTTCTTTTGTGTTGTGTATCTCTGTT	This study
ECs1821-EcoRI-1-us	ATTCATGAATTCTTTTTAGATTCATAGTTTAATGTTTC	This study
ECs1821-SalI-1-ds	ATTCATGTCGACCTGATTTGTTTGTATTATTTGTCTCT	This study
1567-EcoRI-2-us	ATTCATGAATTCAATGCCATTTTCAATCAAAAACAGATTTTCAAG	This study
1567-BamHI-1-ds	ATTCATGGATCCCTATTCTTTTGTGTTGTGTATCTCTGTTTCTATAG	This study
1821-EcoRI-2-us	ATTCATGAATTCAATGCCATTTTCAATAAAAAGTATTTTTTCAGGAC	This study
1821-BamHI-1-ds	ATTCATGGATCCCTATGATTTGTTTGTATTATTTGTCTCTGTC	This study
EcoRI+1567_N-2	GCCGAATTCATGCCATTTTCAATCAAAAAC	This study
1567_C(stop-)+SalI-2	GCCGTCGACTTCTTTTGTGTTGTGTATCTCTG	This study
EcoRI+1821_N-2	GCCGAATTCATGCCATTTTCAATAAAAAG	This study
1821_C(stop-)+SalI-2	GCCGTCGACTGATTTGTTTGTATTATTTGTCTC	This study
Xho+EspM1_N	GCCCTCGAGATGCCAGTAAATGCGACAGGTG	This study
EspM1_C+Bgl2	GCCAGATCTTTACCCCTGTATAACACGAC	This study
Xho+EspM2_N	GCCCTCGAGATGCCGATGAATACTACAGGTA	This study
EspM2_C+Bgl2	GCCAGATCTTCATCCCTGTATAGCACGCA	This study
Xho+MAP_N	GCCCTCGAGATGTTTAGTCCAATGACAATG	This study
MAP_C+Bgl2	GCCAGATCTCTACAATCGGGTATCCTGTAC	This study
Xho+EspG_N	GCCCTCGAGATGATACTTGTTGCCAAATTG	This study
EspG_C+Bgl2	GCCAGATCTTTAAGTGTTTTGTAAGTACG	This study

Plasmids pEspO1-1 and pEspO1-2 were used for complementation analyses. Plasmids pEspO1-1-HA and pEspO1-2-HA were used for infection and translocation assays. Plasmids pRFP-EspO1-1, pRFP-EspO1-2 and pEGFP-EspM2 were used for transfection assays. Plasmids pGEX-5X-1, pGST-EspO1-1, pGST-EspO1-2, pFLAG-MAC, pFLAG-EspM1, pFLAG-EspM2, pFLAG-MAP and pFLAG-EspG were used for GST pull-down assays and binding analyses.

HeLa cells were grown in DMEM with 10% fetal calf serum (FCS) in a 5% CO_2_ incubator.

### Construction of Plasmids

To construct plasmids pEspO1-1 and pEspO1-2, a PCR product containing an intact *espO1-1* or *espO1-2* gene that had been amplified from EHEC genomic DNA with primer set ECs1567-EcoRI-1-us/ECs1567-SalI-2-ds or ECs1821-EcoRI-1-us/ECs1821-SalI-2-ds, respectively, was inserted into the *EcoR*I-*Sal*I sites of pTH18cr. A PCR product containing an intact *espO1-1* or *espO1-2* gene amplified from EHEC genomic DNA with primer set ECs1567-EcoRI-2us/ECs1567-SalI-1'-ds or ECs1821-EcoRI-1-us/ECs1821-SalI-1-ds, respectively, was inserted into the *EcoR*I-*Sal*I sites of pIRES-hrGFP-2a to yield an *espO1-1* or *espO1-2* intact gene fused to a hemagglutinin tag (HA), resulting in pIRES-hrGFP-2a-*espO1-1* and pIRES-hrGFP-2a-*espO1-2,* respectively. To construct plasmids pEspO1-1-HA and pEspO1-2-HA, a PCR product containing *espO1-1*-HA or *espO1-2*-HA amplified from plasmid pIRES-hrGFP-2a-*espO1-1* or pIRES-hrGFP-2a-*espO1-2*, respectively, with primer set IRES-XbaI-ds-1/IRES-PstI-us-1 was inserted into the *Xba*I-*Pst*I sites of pK19. Plasmid pK19HA was constructed by cloning a PCR product amplified from plasmid pIRES-hrGFP-2a with primer set IRES-XbaI-ds-1/IRES-PstI-us-1 into the *Xba*I-*Pst*I sites of pK19. To construct plasmids pREP-EspO1-1 and pREP-EspO1-2, a PCR product containing an intact *espO1-1* or *espO1-2* gene amplified with primer set 1567-EcoRI-2-us/1567-BamHI-1-ds or primer set 1821-EcoRI-2-us/1821-BamHI-1-ds, respectively, was inserted into the *EcoR*I-*Bam*HI sites of pDsRed-monomer C1. To construct plasmids pGST-EspO1-1 and pGST-EspO1-2, a PCR product containing an intact *espO1-1* or *espO1-2* gene amplified with primer set EcoRI+1567_N-2/1567_C(stop-)+SalI-2 or primer set EcoRI+1821_N-2/1821_C(stop-)+SalI-2, respectively, was inserted into the EcoRI-SalI sites of pGEX-5X-1. To construct plasmids pFLAG-EspM1, pFLAG-EspM2, pFLAG-MAP and pFLAG-EspG, a PCR product containing an intact *espM1*, *espM2*, map or *espG* gene amplified with primer set Xho+EspM1_N/EspM1_C+Bgl2, primer set Xho+EspM2_N/EspM2_C+Bgl2, primer set Xho+MAP_N/MAP_C+Bgl2 or primer set Xho+EspG_N/EspG_C+Bgl2, respectively, was inserted into the XhoI-BglII sites of pFLAG-MAC. The structure of each construct was confirmed by DNA sequencing and restriction enzyme analysis.

### Infection and Translocation Assays

Infection and translocation assays were performed as described previously [38], with slight modifications. HeLa cells were inoculated in a 6-well plate (3×10^5^ cells/well) or a 24-well plate (6×10^4^ cells/well) and incubated for 24 h in DMEM in the presence of 5% CO_2_. The cells were then washed three times with serum-free DMEM. EHEC strains cultured overnight at 37°C without shaking in DMEM containing 0.5% glycerol with or without antibiotics were added to the cells in each well at a multiplicity of infection of 100. The plates were centrifuged at 1,000×g for 5 min and then incubated for 1 h at 37°C in the presence of 5% CO_2_. The cells were then washed three times with PBS, incubated for an additional 2 or 3 h in serum-free DMEM, and the cell monolayers were washed three times with PBS. The cells were then fixed with methanol and stained with Giemsa reagent, or fixed with 3.7% paraformaldehyde and stained with appropriate antibodies for immunofluorescence.

### Transfection Assays

HeLa cells were seeded on coverslips in a 24-well plate in DMEM containing 10% FCS. The cells were washed once, incubated with serum-free DMEM for 30 min at 37°C, and then transfected with 1 µg of each DNA construct and 3 µl Lipofectamine 2000 reagent (Invitrogen), in accordance with the manufacturer’s instructions. After 24 h transfection, the cells were incubated in DMEM containing 10% FCS for 1 or 2 d at 37°C. The cells were fixed with 3.7% paraformaldehyde and stained with antibodies appropriate for immunofluorescence.

### Immunofluorescence Staining

Fixed cells were treated for 5 min with 50 mM NH_4_Cl in PBS. The cells were permeabilized with 0.2% Triton X-100 in PBS for 5 min at room temperature and then treated with 2% bovine serum albumin in PBS for 30 min. For immunofluorescence staining, Alexa 488-labeled phalloidin (Molecular Probes) and Alexa 350-labeled phalloidin (Molecular Probes) were used to visualize the actin cytoskeleton. DAPI (Molecular Probes) was used to visualize DNA. Anti-vinculin (Sigma) and anti-HA (Sigma) antibodies were used as primary antibodies. Alexa 488-conjugated anti-mouse IgG (Molecular Probes) and Texas Red conjugated anti-mouse IgG (Molecular Probes) antibodies were used as secondary antibodies to visualize vinculin and HA, respectively. Cells were observed with a confocal laser scanning microscope LSM510 or fluorescence microscope. The number of FAs was analyzed with Scion Image software.

### Production and Preparation of Recombinant Proteins

For GST-fused proteins, *Escherichia coli* BL21 carrying pGEX-5X constructs were cultivated in L-broth supplemented with ampicillin (50 µg/ml) for 3 h at 37°C. Fused protein expression was induced by addition of 1 mM isopropyl 1-thio-d-galactopyranoside (IPTG) and incubation for 3 h at 37°C. Bacteria were disrupted by sonication and GST-fused proteins were purified with glutathione-Sepharose 4B beads (GE Healthcare) according to the manufacturer's protocol.

### GST Pull-down Assays and Binding Analyses

For these experiments, 1.5×10^6^ HeLa cells were inoculated in 10 cm Petri dishes and incubated for 24 h in the presence of 5% CO_2_. The cells were then washed once with PBS, and harvested. *E. coli* MC1061 carrying the pFLAG-MAC construct was cultured in L-broth with 50 µg ampicillin/ml for 2 h at 37°C. IPTG was then added to a final concentration of 1 mM. After incubation for 2 h at 37°C, the bacteria were harvested. The HeLa cells and bacteria were collected and lysed by sonication in 1 ml ice-cold RIPA buffer (25 mM Tris-HCl, 150 mM NaCl, 1% (v/v) Nonidet P-40, 1 mM AEBSF, pH 7.5). The lysates were centrifuged at 15,000×g for 30 min at 4°C, and the supernatants (lysates) were used for pull-down assays and binding analysis.

GST-fused protein constructs bound to 25 µl glutathione-Sepharose 4B beads were mixed with HeLa cell lysates or a cleared extract of MC1061 carrying pFLAG-MAC and incubated for 2 h at 4°C. After centrifugation, the supernatants and beads were washed four times with ice-cold RIPA buffer. After another wash, supernatants were removed and 25 µl SDS-PAGE sample buffer was added to each sample. GST-fused proteins were detected by immunoblotting with anti-ILK (Cell Signaling) and anti-Flag (Sigma) antibodies.

### GST-RBD Pull-down Assays

For these assays, 1.5×10^6^ HeLa cells were inoculated in 10 cm Petri dishes and incubated for 24 h in the presence of 5% CO_2_. The cells were then washed three times with serum-free DMEM and incubated overnight in serum-free DMEM, and EHEC strains that had been cultured overnight at 37°C in DMEM containing 0.5% glycerol were added to the cells at a multiplicity of infection of 100. The culture was incubated for 1 h at 37°C in the presence of 5% CO_2_. The cells were then washed three times with PBS and incubated for an additional 1 or 3 h, after which the monolayers were washed three times with PBS. The cells were then lysed in RIPA buffer and GST-RBD pull-down assays were done in accordance with the manufacturer’s instructions (Cytoskeleton). The amount of RhoA that co-precipitated with GST-RBD and the level of RhoA expression in the whole cell lysates were measured using immunoblotting with anti-RhoA antibodies (Cytoskeleton). The quantified data for band intensities in different sets of experiments was generated by analyzing by the Image J program (Version 1.42, USA).

## Supporting Information

Figure S1 **Formation of actin filaments and focal adhesions (FAs) in HeLa cells infected with EHEC.** HeLa cells were infected with WT, Δ*espO1-1*Δ*espO1-2*, Δ*espO1-1* or Δ*espO1-2*. At 4 h post-infection, the cells were fixed and processed for confocal laser scanning microscopy using rhodamine-phalloidin to visualize actin filaments (red), anti-vinculin antibody to visualize FAs (green), and DAPI to visualize DNA/chromosomes (blue). Images are 2D projections of 5 optical sections spanning 1.0 µm in z-depth. Arrow-heads indicate the characteristic actin condensation beneath the bacteria. Scale bar, 20 µm.
(TIF)

Figure S2 **Interaction of EspO1-1 and EspO1-2 with ILK.** (A) Interaction of EspO1-1 and EspO1-2 with ILK was analyzed by GST pull-down assays with HeLa cells. Proteins bound to GST-EspO1-1, GST-EspO1-2 and GST-alone were analyzed by SDS-PAGE followed by immunoblotting with anti-ILK antibody. (B) Localization of ectopically-expressed EspO1-1 and EspO1-2 in HeLa cells. HeLa cells were transfected with pRFP-EspO1-1 or pRFP-EspO1-2 and immunostained with an anti-vinculin antibody (green) and phallidin (blue). Scale bar, 10 µm. (C) The number of FAs in HeLa cells with ectopically-expressed EspO1-1 and EspO1-2 was visualized using vinculin staining as shown in Fig. S1B and quantified (>10 cells, n = 3). Data are the mean and S.D. **P<0.05, ***P<0.01. (D) To map the EspM2-binding site in EspO1-1 and compare the ability of EspO1-1 and EspO1-2 to interact with EspM2, a series of truncated GST-EspO1-1 peptides was used in binding assays with cell lysate expressing Flag-tagged EspM2.
(TIF)

Figure S3 **Localization of EspO1-2 at FAs in EHEC-infected cells.** (A) HeLa cells were infected with Δ*espM2*Δ*espO1-1*Δ*espO1-2* or Δ*espO1-1*Δ*espO1-2* carrying pEspO1-2-HA. Epithelial cells were infected with these strains for 4 h and immunofluorescence-stained with Alexa 488-conjugated phalloidin (green) and anti-HA antibody (red). (B) The percent of cells with efficient localization of EspO1-2 at FAs shown in (A) was quantified.
(TIF)

Figure S4 **Alignment of the amino-acid sequence of the C-terminal region of OspE homologs in EHEC.** Alignment of the amino-acid sequence of the C-terminal region that corresponds to an EspM2 binding region of OspE homologs in EHEC O157 Sakai, O26 11368 and O111 11128 strains. The amino-acid sequence analysis classified the OspE homologs into two groups, one similar to EspO1-1 and the other similar to EspO1-2. Asterisks and black background indicate an amino acid that is similar to EspO1-1 or EspO1-2, respectively.
(TIF)

## References

[1] Frankel G, Phillips AD, Rosenshine I, Dougan G, Kaper JB, et al (1998) Enteropathogenic and enterohaemorrhagic *Escherichia coli*: more subversive elements. Mol Microbiol 30: 911–921. 9988469 10.1046/j.1365-2958.1998.01144.x

[2] Nataro JP, Kaper JB (1998) Diarrheagenic *Escherichia coli* . Clin Microbiol Rev 11: 142–201. 9457432 10.1128/cmr.11.1.142 PMC121379

[3] Kaper JB, Nataro JP, Mobley HL (2004) Pathogenic *Escherichia coli* . Nat Rev Microbiol 2: 123–140. 15040260 10.1038/nrmicro818

[4] Knutton S, Baldwin T, Williams PH, McNeish AS (1989) Actin accumulation at sites of bacterial adhesion to tissue culture cells: basis of a new diagnostic test for enteropathogenic and enterohemorrhagic *Escherichia coli* . Infect Immun 57: 1290–1298. 2647635 10.1128/iai.57.4.1290-1298.1989 PMC313264

[5] Hayward RD, Leong JM, Koronakis V, Campellone KG (2006) Exploiting pathogenic *Escherichia coli* to model transmembrane receptor signalling. Nat Rev Microbiol 4: 358–370. 16582930 10.1038/nrmicro1391

[6] Vingadassalom D, Kazlauskas A, Skehan B, Cheng HC, Magoun L, et al (2009) Insulin receptor tyrosine kinase substrate links the *E. coli* O157:H7 actin assembly effectors Tir and EspF(U) during pedestal formation. Proc Natl Acad Sci U S A 106: 6754–6759. 19366662 10.1073/pnas.0809131106 PMC2672544

[7] Weiss SM, Ladwein M, Schmidt D, Ehinger J, Lommel S, et al (2009) IRSp53 links the enterohemorrhagic *E. coli* effectors Tir and EspFU for actin pedestal formation. Cell Host Microbe 5: 244–258. 19286134 10.1016/j.chom.2009.02.003

[8] Tobe T, Beatson SA, Taniguchi H, Abe H, Bailey CM, et al (2006) An extensive repertoire of type III secretion effectors in *Escherichia coli* O157 and the role of lambdoid phages in their dissemination. Proc Natl Acad Sci U S A 103: 14941–14946. 16990433 10.1073/pnas.0604891103 PMC1595455

[9] Mellies JL, Barron AM, Carmona AM (2007) Enteropathogenic and enterohemorrhagic *Escherichia coli* virulence gene regulation. Infect Immun 75: 4199–4210. 17576759 10.1128/IAI.01927-06 PMC1951183

[10] Deng W, Puente JL, Gruenheid S, Li Y, Vallance BA, et al (2004) Dissecting virulence: systematic and functional analyses of a pathogenicity island. Proc Natl Acad Sci U S A 101: 3597–3602. 14988506 10.1073/pnas.0400326101 PMC373508

[11] McDaniel TK, Jarvis KG, Donnenberg MS, Kaper JB (1995) A genetic locus of enterocyte effacement conserved among diverse enterobacterial pathogens. Proc Natl Acad Sci U S A 92: 1664–1668. 7878036 10.1073/pnas.92.5.1664 PMC42580

[12] Hall A (1998) Rho GTPases and the actin cytoskeleton. Science 279: 509–514. 9438836 10.1126/science.279.5350.509

[13] Etienne-Manneville S, Hall A (2002) Rho GTPases in cell biology. Nature 420: 629–635. 12478284 10.1038/nature01148

[14] Heasman SJ, Ridley AJ (2008) Mammalian Rho GTPases: new insights into their functions from in vivo studies. Nat Rev Mol Cell Biol 9: 690–701. 18719708 10.1038/nrm2476

[15] Tybulewicz VL, Henderson RB (2009) Rho family GTPases and their regulators in lymphocytes. Nat Rev Immunol 9: 630–644. 19696767 10.1038/nri2606 PMC4898593

[16] Parsons JT, Horwitz AR, Schwartz MA (2010) Cell adhesion: integrating cytoskeletal dynamics and cellular tension. Nat Rev Mol Cell Biol 11: 633–643. 20729930 10.1038/nrm2957 PMC2992881

[17] Alto NM, Shao F, Lazar CS, Brost RL, Chua G, et al (2006) Identification of a bacterial type III effector family with G protein mimicry functions. Cell 124: 133–145. 16413487 10.1016/j.cell.2005.10.031

[18] Arbeloa A, Bulgin RR, MacKenzie G, Shaw RK, Pallen MJ, et al (2008) Subversion of actin dynamics by EspM effectors of attaching and effacing bacterial pathogens. Cell Microbiol 10: 1429–1441. 18331467 10.1111/j.1462-5822.2008.01136.x PMC2610399

[19] Bulgin R, Raymond B, Garnett JA, Frankel G, Crepin VF, et al (2010) Bacterial guanine nucleotide exchange factors SopE-like and WxxxE effectors. Infect Immun 78: 1417–1425. 20123714 10.1128/IAI.01250-09 PMC2849395

[20] Matsuzawa T, Kuwae A, Yoshida S, Sasakawa C, Abe A (2004) Enteropathogenic *Escherichia coli* activates the RhoA signaling pathway via the stimulation of GEF-H1. EMBO J 23: 3570–3582. 15318166 10.1038/sj.emboj.7600359 PMC516631

[21] Kim M, Ogawa M, Fujita Y, Yoshikawa Y, Nagai T, et al (2009) Bacteria hijack integrin-linked kinase to stabilize focal adhesions and block cell detachment. Nature 459: 578–582. 19489119 10.1038/nature07952

[22] Arbeloa A, Garnett J, Lillington J, Bulgin RR, Berger CN, et al (2010) EspM2 is a RhoA guanine nucleotide exchange factor. Cell Microbiol 12: 654–664. 20039879 10.1111/j.1462-5822.2009.01423.x PMC2871174

[23] Simovitch M, Sason H, Cohen S, Zahavi EE, Melamed-Book N, et al (2010) EspM inhibits pedestal formation by enterohaemorrhagic *Escherichia coli* and enteropathogenic *E. coli* and disrupts the architecture of a polarized epithelial monolayer. Cell Microbiol 12: 489–505. 19912240 10.1111/j.1462-5822.2009.01410.x

[24] Ridley AJ, Schwartz MA, Burridge K, Firtel RA, Ginsberg MH, et al (2003) Cell migration: integrating signals from front to back. Science 302: 1704–1709. 14657486 10.1126/science.1092053

[25] Deng W, de Hoog CL, Yu HB, Li Y, Croxen MA, et al (2010) A comprehensive proteomic analysis of the type III secretome of Citrobacter rodentium. J Biol Chem 285: 6790–6800. 20034934 10.1074/jbc.M109.086603 PMC2825473

[26] Dong N, Liu L, Shao F (2010) A bacterial effector targets host DH-PH domain RhoGEFs and antagonizes macrophage phagocytosis. EMBO J 29: 1363–1376. 20300064 10.1038/emboj.2010.33 PMC2868573

[27] Bershadsky A, Chausovsky A, Becker E, Lyubimova A, Geiger B (1996) Involvement of microtubules in the control of adhesion-dependent signal transduction. Curr Biol 6: 1279–1289. 8939572 10.1016/s0960-9822(02)70714-8

[28] Chrzanowska-Wodnicka M, Burridge K (1996) Rho-stimulated contractility drives the formation of stress fibers and focal adhesions. J Cell Biol 133: 1403–1415. 8682874 10.1083/jcb.133.6.1403 PMC2120895

[29] Pelham RJ Jr, Wang Y (1997) Cell locomotion and focal adhesions are regulated by substrate flexibility. Proc Natl Acad Sci U S A 94: 13661–13665. 9391082 10.1073/pnas.94.25.13661 PMC28362

[30] Helfman DM, Levy ET, Berthier C, Shtutman M, Riveline D, et al (1999) Caldesmon inhibits nonmuscle cell contractility and interferes with the formation of focal adhesions. Mol Biol Cell 10: 3097–3112. 10512853 10.1091/mbc.10.10.3097 PMC25564

[31] Kaverina I, Krylyshkina O, Small JV (1999) Microtubule targeting of substrate contacts promotes their relaxation and dissociation. J Cell Biol 146: 1033–1044. 10477757 10.1083/jcb.146.5.1033 PMC2169483

[32] Ridley AJ, Hall A (1992) The small GTP-binding protein rho regulates the assembly of focal adhesions and actin stress fibers in response to growth factors. Cell 70: 389–399. 1643657 10.1016/0092-8674(92)90163-7

[33] Broussard JA, Webb DJ, Kaverina I (2008) Asymmetric focal adhesion disassembly in motile cells. Curr Opin Cell Biol 20: 85–90. 18083360 10.1016/j.ceb.2007.10.009

[34] Gupton SL, Waterman-Storer CM (2006) Spatiotemporal feedback between actomyosin and focal-adhesion systems optimizes rapid cell migration. Cell 125: 1361–1374. 16814721 10.1016/j.cell.2006.05.029

[35] Ogura Y, Ooka T, Iguchi A, Toh H, Asadulghani M, et al (2009) Comparative genomics reveal the mechanism of the parallel evolution of O157 and non-O157 enterohemorrhagic *Escherichia coli* . Proc Natl Acad Sci U S A 106: 17939–17944. 19815525 10.1073/pnas.0903585106 PMC2764950

[36] Hayashi T, Makino K, Ohnishi M, Kurokawa K, Ishii K, et al (2001) Complete genome sequence of enterohemorrhagic *Escherichia coli* O157:H7 and genomic comparison with a laboratory strain K-12. DNA Res 8: 11–22. 11258796 10.1093/dnares/8.1.11

[37] Datsenko KA, Wanner BL (2000) One-step inactivation of chromosomal genes in *Escherichia coli* K-12 using PCR products. Proc Natl Acad Sci U S A 97: 6640–6645. 10829079 10.1073/pnas.120163297 PMC18686

[38] Iyoda S, Koizumi N, Satou H, Lu Y, Saitoh T, et al (2006) The GrlR-GrlA regulatory system coordinately controls the expression of flagellar and LEE-encoded type III protein secretion systems in enterohemorrhagic *Escherichia coli* . J Bacteriol 188: 5682–5692. 16885436 10.1128/JB.00352-06 PMC1540053

[39] Kenny B, DeVinney R, Stein M, Reinscheid DJ, Frey EA, et al (1997) Enteropathogenic *E. coli* (EPEC) transfers its receptor for intimate adherence into mammalian cells. Cell 91: 511–520. 9390560 10.1016/s0092-8674(00)80437-7

[40] Khyrul WA, LaLonde DP, Brown MC, Levinson H, Turner CE (2004) The integrin-linked kinase regulates cell morphology and motility in a rho-associated kinase-dependent manner. J Biol Chem 279: 54131–54139. 15485819 10.1074/jbc.M410051200

