# Supplementary figures and images for: EspO1-2 Regulates EspM2-Mediated RhoA Activity to Stabilize Formation of Focal Adhesions in Enterohemorrhagic Escherichia coli-Infected Host Cells
Source: PLoS One. 2013 Feb 8;8(2):e55960. doi: 10.1371/journal.pone.0055960 (PMC3568036; doi:10.1371/journal.pone.0055960)

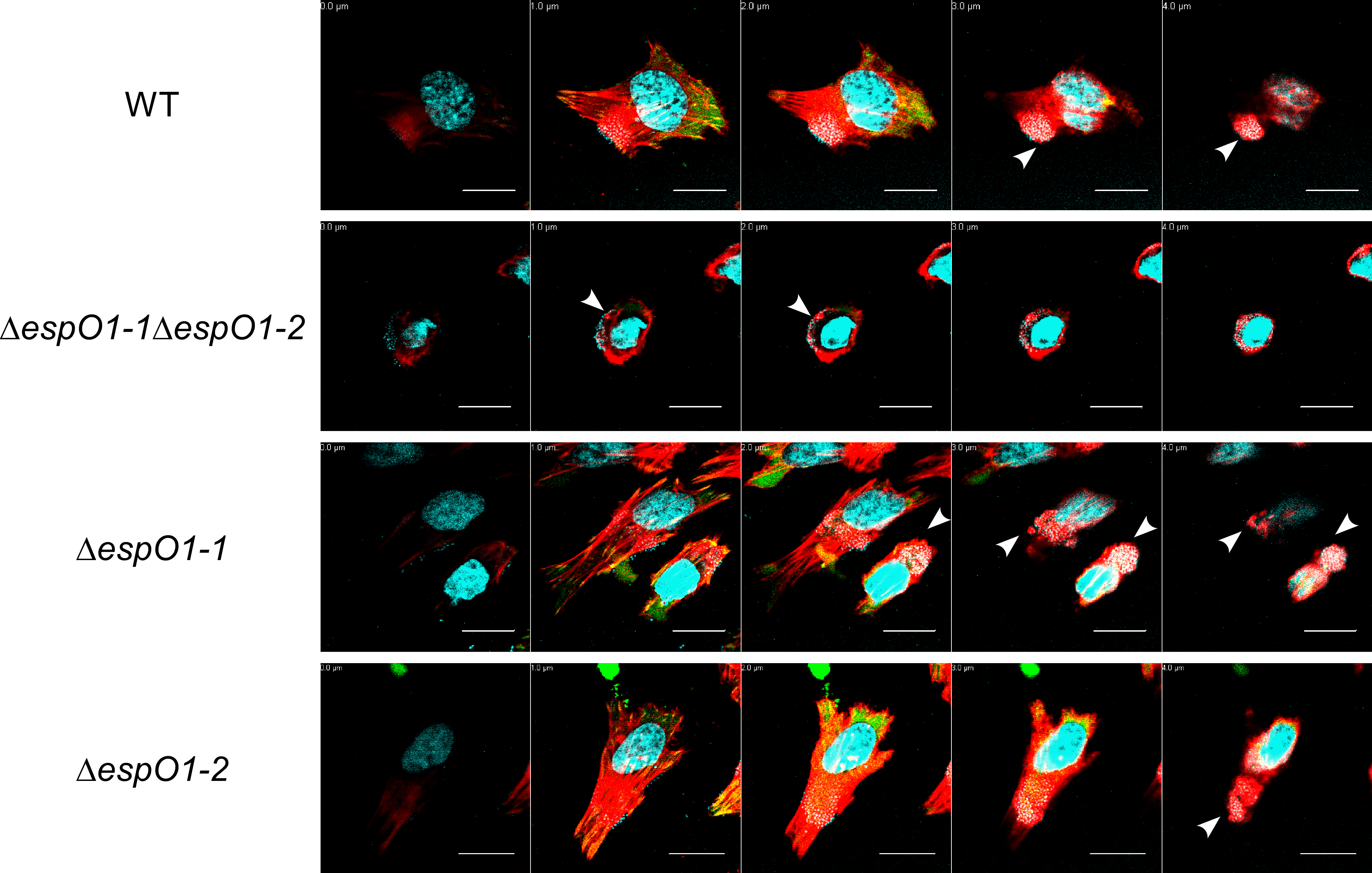

Supplement: Figure S1 — Formation of actin filaments and focal adhesions (FAs) in HeLa cells infected with EHEC. HeLa cells were infected with WT, ΔespO1-1ΔespO1-2, ΔespO1-1 or ΔespO1-2. At 4 h post-infection, the cells were fixed and processed for confocal laser scanning microscopy using rhodamine-phalloidin to visualize actin filaments (red), anti-vinculin antibody to visualize FAs (green), and DAPI to visualize DNA/chromosomes (blue). Images are 2D projections of 5 optical sections spanning 1.0 µm in z-depth. Arrow-heads indicate the characteristic actin condensation beneath the bacteria. Scale bar, 20 µm. (TIF) [file pone.0055960.s001.tif]

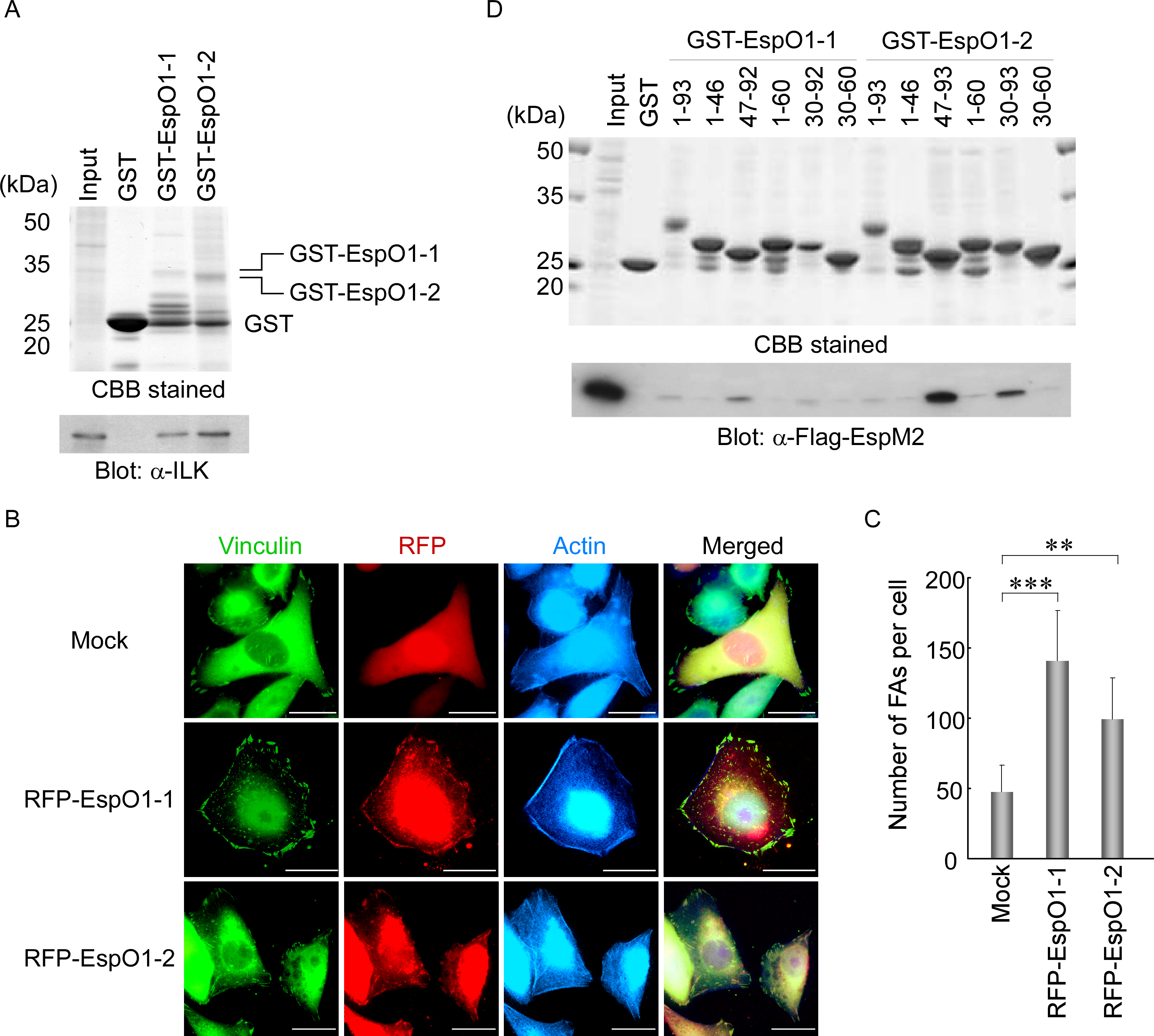

Supplement: Figure S2 — Interaction of EspO1-1 and EspO1-2 with ILK. (A) Interaction of EspO1-1 and EspO1-2 with ILK was analyzed by GST pull-down assays with HeLa cells. Proteins bound to GST-EspO1-1, GST-EspO1-2 and GST-alone were analyzed by SDS-PAGE followed by immunoblotting with anti-ILK antibody. (B) Localization of ectopically-expressed EspO1-1 and EspO1-2 in HeLa cells. HeLa cells were transfected with pRFP-EspO1-1 or pRFP-EspO1-2 and immunostained with an anti-vinculin antibody (green) and phallidin (blue). Scale bar, 10 µm. (C) The number of FAs in HeLa cells with ectopically-expressed EspO1-1 and EspO1-2 was visualized using vinculin staining as shown in Fig. S1B and quantified (>10 cells, n = 3). Data are the mean and S.D. **P<0.05, ***P<0.01. (D) To map the EspM2-binding site in EspO1-1 and compare the ability of EspO1-1 and EspO1-2 to interact with EspM2, a series of truncated GST-EspO1-1 peptides was used in binding assays with cell lysate expressing Flag-tagged EspM2. (TIF) [file pone.0055960.s002.tif]

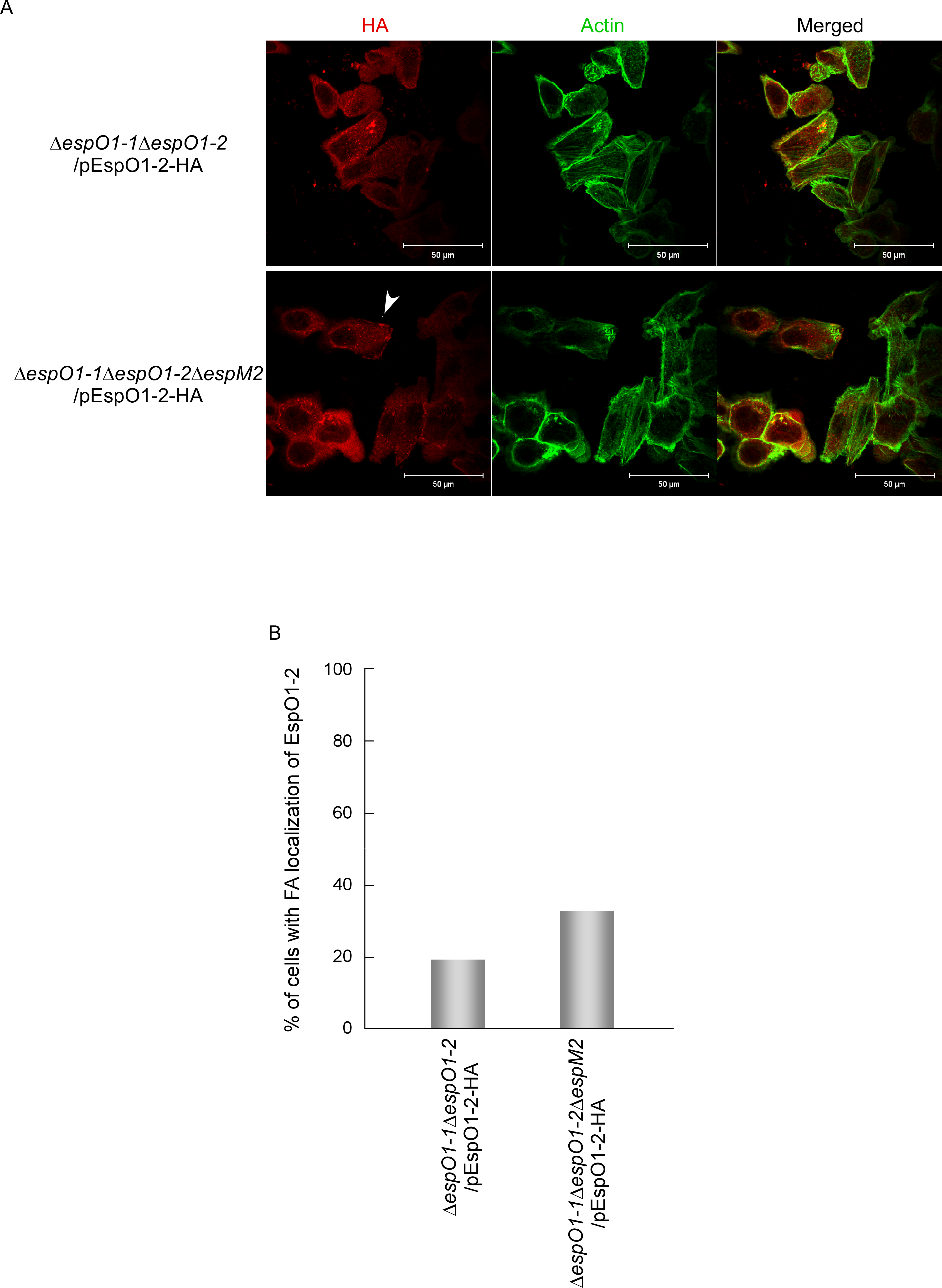

Supplement: Figure S3 — Localization of EspO1-2 at FAs in EHEC-infected cells. (A) HeLa cells were infected with ΔespM2ΔespO1-1ΔespO1-2 or ΔespO1-1ΔespO1-2 carrying pEspO1-2-HA. Epithelial cells were infected with these strains for 4 h and immunofluorescence-stained with Alexa 488-conjugated phalloidin (green) and anti-HA antibody (red). (B) The percent of cells with efficient localization of EspO1-2 at FAs shown in (A) was quantified. (TIF) [file pone.0055960.s003.tif]

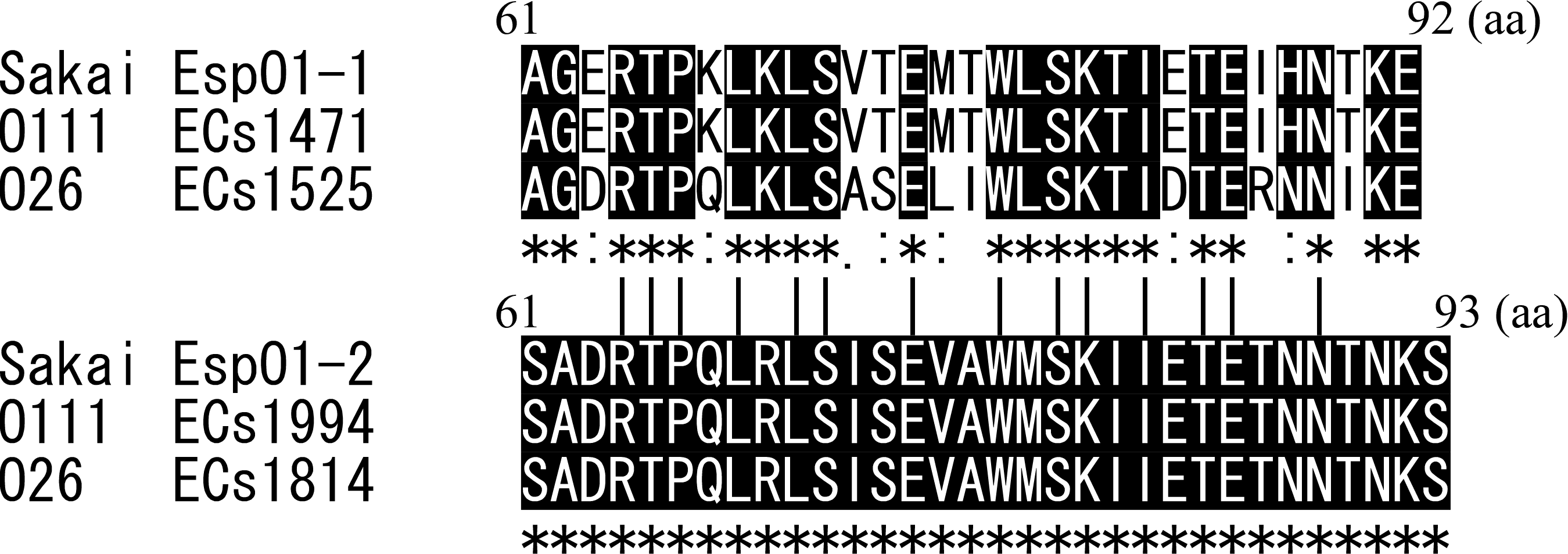

Supplement: Figure S4 — Alignment of the amino-acid sequence of the C-terminal region of OspE homologs in EHEC. Alignment of the amino-acid sequence of the C-terminal region that corresponds to an EspM2 binding region of OspE homologs in EHEC O157 Sakai, O26 11368 and O111 11128 strains. The amino-acid sequence analysis classified the OspE homologs into two groups, one similar to EspO1-1 and the other similar to EspO1-2. Asterisks and black background indicate an amino acid that is similar to EspO1-1 or EspO1-2, respectively. (TIF) [file pone.0055960.s004.tif]
